# Structural ontogeny of protein-protein interactions

**DOI:** 10.1126/science.adx6931

**Published:** 2026-02-12

**Authors:** Aerin Yang, Hanlun Jiang, Kevin M. Jude, Deniz Akpinaroglu, Stephan Allenspach, Alex Jie Li, James Bowden, Carla Patricia Perez, Liu Liu, Po-Ssu Huang, Tanja Kortemme, Jennifer Listgarten, K. Christopher Garcia

**Affiliations:** 1 Department of Molecular and Cellular Physiology, Stanford University School of Medicine, Stanford, CA 94305, USA.; 2 Department of Electrical Engineering and Computer Science, UC Berkeley, CA, USA.; 3 Center for Computational Biology, University of California Berkeley, CA, USA; 4 Howard Hughes Medical Institute, Stanford University School of Medicine, Stanford, CA 94305, USA.; 5 The UC Berkeley–UCSF Graduate Program in Bioengineering, University of California, San Francisco; San Francisco, CA 94143, USA.; 6 Department of Bioengineering and Therapeutic Sciences, University of California, San Francisco; San Francisco, CA 94143, USA.; 7 Biophysics program, Stanford University, Stanford, CA, USA; 8 Department of Bioengineering, Stanford University, Stanford, CA, USA.; 9 Quantitative Biosciences Institute, University of California, San Francisco; San Francisco, CA 94143, USA.

## Abstract

Understanding how protein binding sites evolve interactions with other proteins could hold clues to targeting “undruggable” surfaces. We used synthetic coevolution to engineer new interactions between naïve surfaces, simulating the *de novo* formation of protein complexes. We isolated seven distinct structural families of protein Z-domain complexes and found that synthetic complexes explore multiple shallow energy wells through ratchet-like docking modes, whereas complexes formed by natural binding sites converged in a deep energy well with a relatively fixed geometry. Epistasis analysis of a machine learning-estimated fitness landscape revealed ‘seed’ contacts between binding partners that anchored the earliest stages of encounter complex formation. Our results suggest that ‘silent’ surfaces have a shallower energy landscape than natural binding sites, disfavoring tight binding, likely due to evolutionary counter-selection.

How new protein-protein interactions arise, diversify, and evolve distinct specificities remains poorly understood. Yet it is an important problem to better understand, as there appear to be distinctive properties of protein surfaces that have evolved to form protein-protein interfaces, versus non-interacting surfaces of proteins (1-3). Natural protein binding sites are usually the most “druggable” sites on a given protein and tend to attract the majority of binders from combinatorial peptide or antibody libraries (4-6). Are there biophysical properties that distinguish regions of protein surfaces that have not evolved to bind to a ligand from natural binding sites? Although it has been proposed that protein binding sites tend to have more exposed hydrophobic amino acids, no definitive rules exist (2, 7, 8). Indeed protein binding sites are characterized by a vast diversity of chemistries and conformations (9-11). As a result, it remains challenging to identify an unknown binding site solely from inspection of an unbound structure (3).

Protein interfaces often evolve under selective pressures, giving rise to compensatory mutations that confer binding specificity and stability synonymous with particular functions (12-15). Coevolution, characterized by reciprocal changes between interacting partners, reflects structural and functional constraints imposed by evolution and provides insights into the ontogeny of protein-protein interactions (16, 17). Early studies have revealed that residues in close physical proximity at protein interfaces tend to coevolve, exhibiting correlated mutations to maintain structural compatibility and functional integrity (18-20). These coevolutionary signals have been leveraged through computational approaches, such as statistical energy models, to infer residue-residue contacts and identify structural features of PPIs (16, 21-23). Although such models effectively infer evolutionary constraints from sequence variation, the data on which they are trained may not capture the full range of coevolutionary trajectories in PPI evolution. Consequently, experimental systems can help us to gain mechanistic insights while exploring areas of complex formation not uncovered by nature alone. Such experiments can directly track coevolutionary dynamics and trajectories, including transient intermediates, under controlled selection (13, 14, 24-26).

We previously developed a synthetic coevolution platform that enables bidirectional coevolution of interacting proteins though “library-on-library” selections, providing a synthetic proxy of protein coevolution at a systems level (27). This experiment demonstrated that coevolution can structurally remodel naturally conserved interaction surfaces into a plethora of diverse interface chemistries and fine specificities through amino acid side chain repacking. Interestingly, however, the remodeled interfaces were constrained into a common docking mode. This observation raised the question of whether these complexes were trapped in a deep energy minimum and thus encumbered with an evolutionary imprint for binding in a stereotyped manner that could not be traversed even with highly diverse libraries. The coevolution platform offers the possibility to address this question by engineering bidirectional interactions between two naïve protein surfaces unencumbered by a natural binding imprint and to study the epistasis and fitness landscapes (28-30), as a proxy for protein-protein evolution.

In this study we ask if there are fundamental differences between surfaces of proteins that have evolved to bind to proteins versus surfaces that have not? We have learned that the type of epistasis, and structural adaptation that shape specificity and orthogonality differ between natural and non-natural protein binding sites. Further, *in silico* epistasis analysis identified initiating “seed” sequences at the earliest stages of an evolutionary path to a high affinity complex.

## Results

### Designing *de novo* interfaces using synthetic coevolution

**Figure 1.**
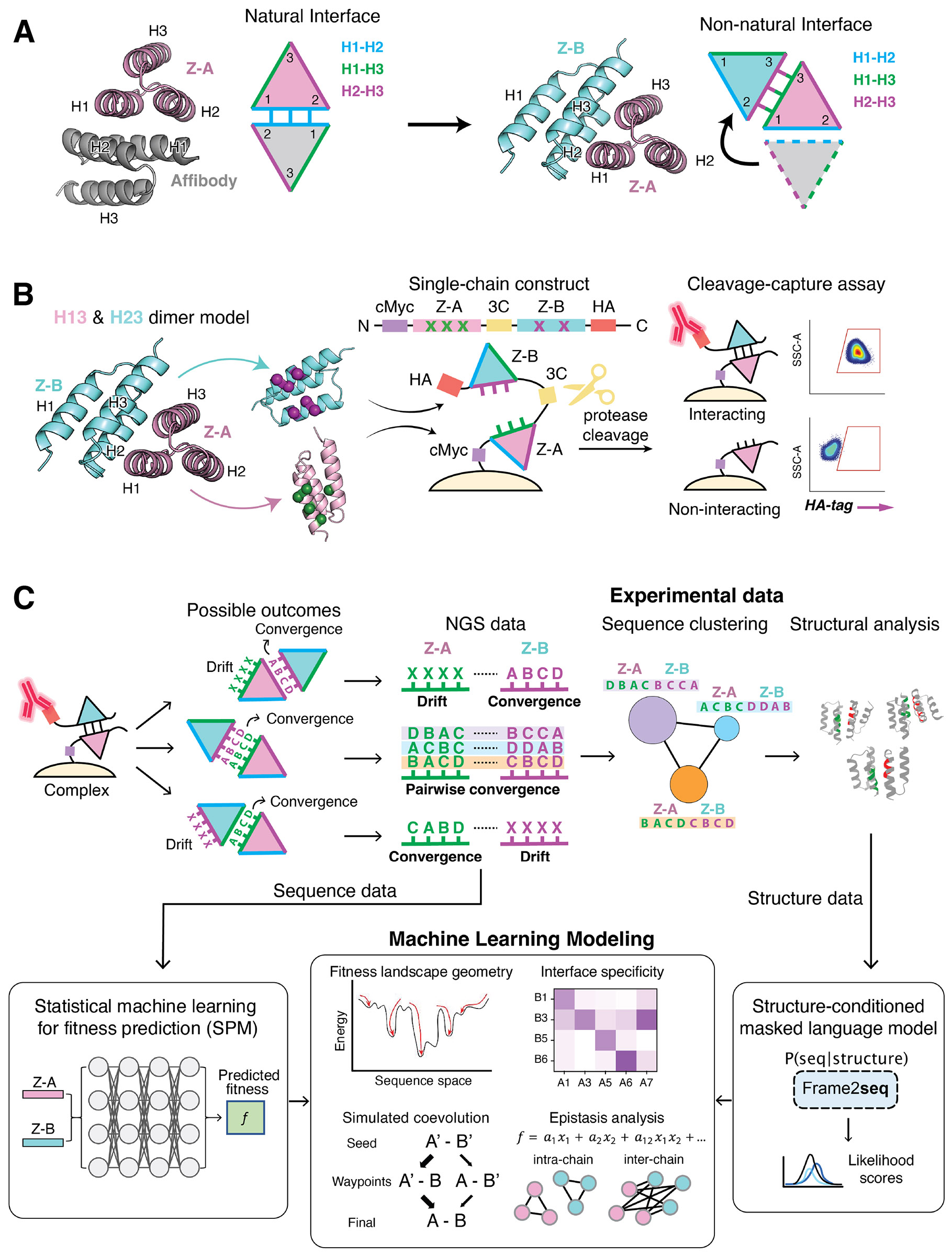
Synthetic coevolution workflow. (A) Overview of the interface relocation strategy. Canonical binding interfaces (H1-H2) of protein Z-domain and affibody were relocated to non-canonical H2-H3 and H1-H3 interfaces to create novel synthetic interfaces. (B) Schematic of the protease-based cleavage-capture assay. Two proteins are displayed on yeast as a single-chain construct connected by a flexible linker containing a 3C protease cleavage site. Upon cleavage, interacting pairs retain fluorescence from the C-terminal HA-tag binding antibody, while non-interacting pairs lose the HA signal, enabling differentiation based on binding affinity. Flow cytometry plots were gated using SSC-A (side scatter area, a measure of cell granularity/complexity) and APC-A (HA-tag fluorescence readout). (C) The workflow integrates experimental and computational approaches. Yeast display screening followed by next-generation sequencing (NGS) is used to track sequence evolution, identify pairwise convergence, and construct sequence similarity networks (SSNs) that visualize cluster formation based on sequence relationships. Crystal structures validate these clusters, revealing interface architecture and binding modes. The NGS data is also harnessed by our statistical machine learning method to model multi-round selection data to obtain an estimated fitness landscape, which can be used to uncover landscape geometry via simulating coevolutionary trajectories, and to elucidate key protein-protein interactions through epistasis analysis. Frame2seq provides sequence-structure mapping, linking sequence clusters to their structural configurations, using crystal structures as input. Epistasis analysis, derived from these computational models and validated by interface structures, reveals critical residue-residue interactions, providing insights into the evolutionary mechanisms shaping protein-protein interactions.

We carried out a synthetic proxy of protein-protein coevolution by designing interfaces between “silent” surfaces of proteins that have no ligand recognition history. We previously described a synthetic coevolution platform that enables bidirectional sampling of libraries to remodel the existing dimer interfaces between the natural binding surface of protein Z-domain and its affibody binder (27). Here, we leveraged this coevolution platform to isolate completely synthetic interfaces between protein Z-domain pairs that have no pre-existing interaction with one another. Our strategy was to “relocate” the binding interface from the canonical binding region that Z-domain uses to bind to its natural ligand, Fc-region of immunoglobulin, as well as synthetic proteins like affibodies, to a non-canonical site on both protein partners that is not used for ligand binding (Fig. 1A). Protein Z-domains and their engineered binding scaffolds, affibodies, typically interact with target proteins through their H1-H2 helices, which we refer to as the “natural” binding sites (31). However, we simultaneously diversified the non-canonical H2-H3 and H1-H3 regions of two Z-domains to encourage these libraries to converge on one another at sites on the proteins that are not used for binding ligands, which we refer to as “synthetic interfaces.” When accompanied by deep sequencing of the selection rounds, this strategy captures the emergence and coevolutionary trajectories of new protein-protein interactions as they transition from unbiased exploratory states to structurally optimized conformations. This approach does not recapitulate the temporal trajectory of natural evolution as all mutants are contained in the initial library (i.e. at time zero prior to any selection), but stepwise selection with higher stringency can simulate, in some respects, affinity maturation over time.

We used our coevolution platform with a protease-based cleavage-capture assay (Fig. 1B). The assay distinguishes between binding and non-binding pairs based on fluorescence retention; interacting pairs displayed on yeast retain HA-tag fluorescence after 3C protease cleavage, whereas non-interacting pairs lose the signal. The HA-tag signal correlates with binding affinities (27), enabling relative comparisons of evolved complexes.

We functionalized the two pre-selected sites of interaction with diverse libraries. Newly coevolved interfaces were revealed by tracking pairwise next-generation sequencing (NGS) sequence information of interacting pairs, uncovering patterns of convergence indicative of coevolution through epistasis. We considered several possible outcomes (Fig. 1C): 1- Sequence clusters showing linkage between Z-A and Z-B sequences indicate successful library-on-library convergence to form a new bidirectional interface 2- Sequence *convergence* in the partner Z-A site paired with sequence *drift* in the Z-B site indicates that Z-A is adapting to an invariant surface on Z-B, and that Z-B sequence drifts because of no selective pressure to adapt to a surface of Z-A, allowing random amino acids. 3- Drift in the Z-A library paired with convergence in the Z-B library indicates Z-A has no interactions, while Z-B has found an invariant surface on Z-A.

We then leveraged machine learning (ML) and statistical modeling to glean more from our experimental data, helping to strengthen our understanding of sequence-structure relationships, and epistatic interactions within them. Specifically, we employed our Selection Probabilistic Model (SPM), a statistical machine learning framework, to extract energy and fitness landscapes from the multi-round selection read count data, capturing both round-specific and global fitness landscapes, encapsulated in a neural network (Methods; fig. S1). Using the global fitness landscape, we simulated *in silico* coevolutionary trajectories, identifying potential evolutionary pathways toward accessible energy minima. In addition, we exploited structural information by using a newly-developed inverse folding model, Frame2seq (32), to comprehensively map the sequence-structure compatibility of all 714 sequences in the selected clusters and the six converged structural configurations with solved crystal structures (Methods). Finally, by performing an epistasis analysis of the SPM energy landscape and the Frame2seq sequence-structure compatibility scores, we uncovered key residue interactions that shaped the ontogeny of our protein-protein interactions.

### Construction and selection of a coevolutionary library for evolving novel protein interfaces

**Figure 2.**
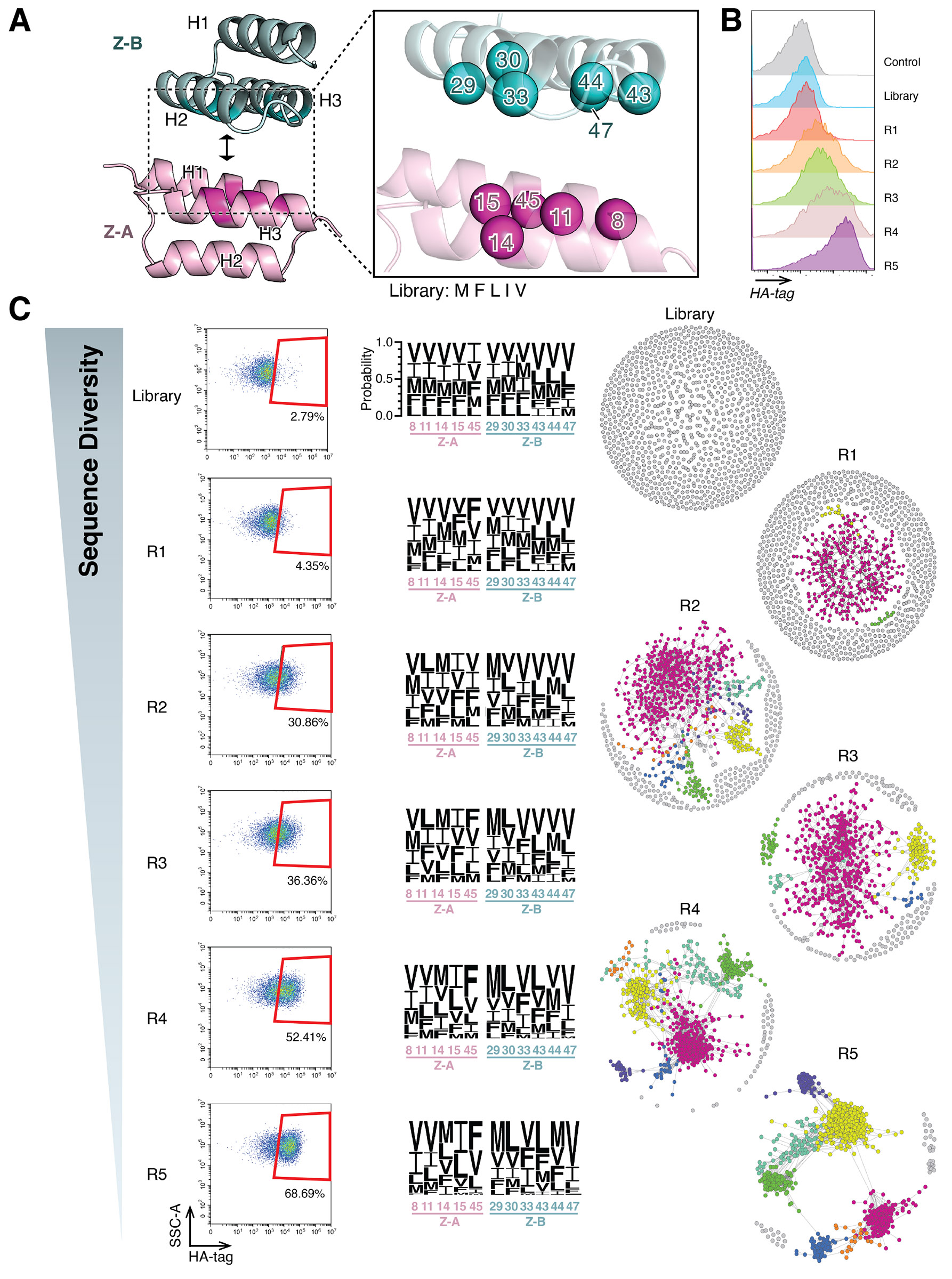
Coevolution selection progress. (A) Coevolution library design targeting non-canonical binding regions (H1-H3 face of Z-A and H2-H3 face of Z-B) in the Z-domain. Eleven library positions were selected for randomization using five hydrophobic amino acids (M, F, L, I, and V). (B) Flow cytometry tracking HA-tag fluorescence enrichment. HA-tag fluorescence was monitored across selection rounds, showing a progressive increase in HA-tag signal, indicative of enrichment for interacting pairs. (C) Selection progression and sequence diversity analysis. (Left) Flow cytometry dot plots demonstrate HA-tag fluorescence enrichment across rounds, with a higher percentage of cells retaining fluorescence in later rounds. (Middle) Sequence logos generated from next-generation sequencing (NGS) data illustrate that amino acid diversity is maintained across all library positions, even in the final round. (Right) Sequence similarity networks (SSNs) of enriched sequences (p-value < 0.05) highlight the emergence of distinct clusters in later rounds, reflecting patterns of convergence at the level of sequence communities rather than individual residues. SSN constructed from concatenated 11 amino acid sequences of Z-A and Z-B proteins. Edges in the SSNs were formed using an edit distance threshold of 3.

To construct our coevolutionary library, we first screened random Z-domain docking interactions to triage for sterically incompatible surface contours, and identify complementary surfaces that might support a new interaction, using Rosetta and PatchDock (33). This process involved docking two poly-valine Z-domains to identify docking poises free of clashes, and that show high shape complementarity, with interface areas exceeding 1000 Å^2^. Using valines as a generic sidechain representation has been widely used in protein design backbone modeling tasks before amino acid identities are resolved (34, 35). Here, in docking the two domains, the valines provided a neutral volume in the initial orientation to define the residues in the interface. Selected docked models underwent iterative refinement and energy minimization in Rosetta, yielding a stable, low-energy H2-H3/H1-H3 poly-Val docking model that served as the basis for our library design (Fig. 2A and fig. S2). Based on the docked poly-Val model, we identified key interface residues for randomization, replacing bulky or charged residues to facilitate efficient contacts between proteins at designated library positions (fig. S3). Five positions (8, 11, 14, 15, and 45) on Z-A and six positions (29, 30, 33, 43, 44, and 47) on Z-B were selected for randomization, using five hydrophobic amino acids (M, F, L, I, and V) to construct the coevolutionary library (Fig. 2A) (36-38). The library was then screened using our coevolution platform, with HA-tag fluorescence monitored by flow cytometry to assess enrichment across rounds (Fig. 2B).

Flow cytometry data revealed progressive enrichment of interacting pairs, as indicated by increased HA-tag signal retention over successive rounds (Fig. 2C left). Selection was carried out until the majority of cells showed clear HA-tag retention after cleavage, relative to pre-cleavage staining, ensuring that libraries were enriched to comparable functional endpoints. Despite this enrichment, sequence logos indicated substantial diversity within the library, without clear convergence to a single consensus sequence even in the later rounds (Fig. 2C middle). This suggests that the library preserved multiple viable configurations rather than converging on a single optimal solution. Notably, sequence similarity networks (SSN) (39) of the top enriched sequences from each round revealed the emergence of distinct clusters in the later rounds, highlighting patterns of convergence at the level of sequence clusters rather than individual residues (Fig. 2C, right). As later analyses indicate, these clusters represent distinct interaction modes evolved at the interface, revealing that the synthetic coevolution platform has captured multiple binding solutions.

### Divergent evolution of synthetic interfaces captured through sequence clustering, structures, and fitness landscape

**Figure 3.**
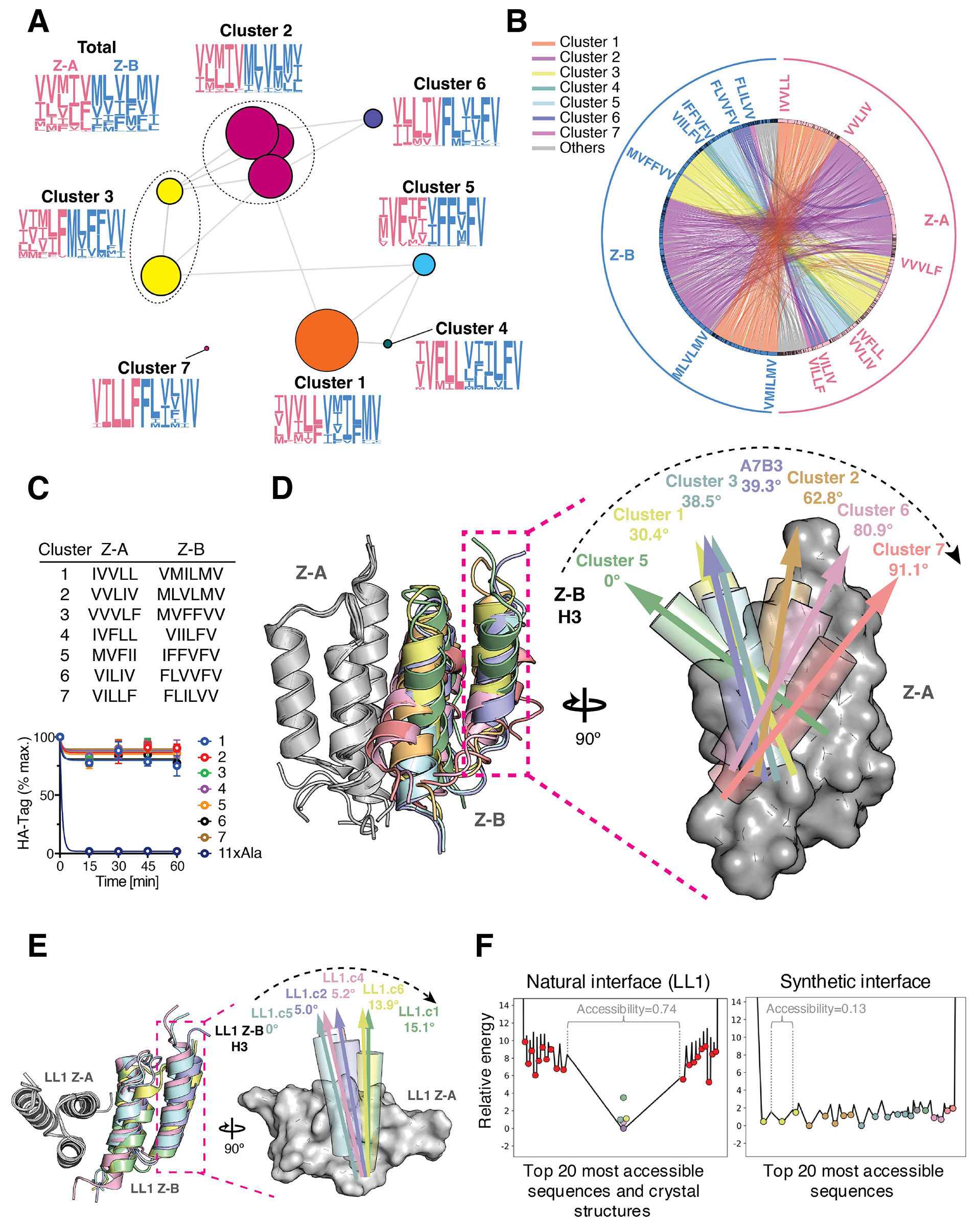
Sequence clustering and fitness landscape of synthetic interfaces. (A) A community map of sequence similarity network (SSN) of round 5 sequences. Clustered communities are merged into single nodes to form a community map. Seven distinct clusters were identified, each exhibiting distinct patterns of convergence, as illustrated in the sequence logos. (B) A Circos plot (40) representing pairwise relationships between Z-A and Z-B proteins in round 5 next-generation sequencing (NGS) data. Each pair is normalized to have equal area, providing a visual representation of the approximate specificity or cross-reactivity of each sequence. The plot highlights high orthogonality, with sequences primarily pairing within their respective clusters and minimal cross-cluster interactions. (C) Validation of representative interactions. (top) A table of representative pairs from each cluster. (Bottom) On-yeast cleavage-capture assay confirms strong binding affinities for all tested pairs, validating functional interactions. Data are mean ± SD; n = 3 independent replicates. (D) Structural diversity in synthetic interface docking orientations. (Left) Superimposed crystal structures of synthetic interface complexes. (Right) Docking angle variations of Z-B helix 3 relative to the Cluster 5 structure highlight the diversity in docking geometries among clusters. (E) Conserved docking orientations in natural interface complexes. Structures from a previous study (27) reveal that natural interface complexes of the Z-domain and its affibody binder exhibit relatively consistent docking angles, in contrast to the synthetic interface complexes in panel D. (F) The relative energy landscapes of the 20 most accessible wells for the natural interface (LL1 library) and the synthetic interface. Energy is defined as the negative fitness (47), and, for ease of comparison of the interfaces, for each interface, we computed a *relative energy* for each sequence by subtracting the energy of the strongest-binding sequence. We do so because relative energies are comparable between the two plots (see Section C of Supplementary Information for details), whereas energies are not. The width of each energy well corresponds to the accessibility (see main text and Methods) of that well. In each subplot, the most accessible energy well is marked by the curly brace labeled with its accessibility. For the natural interface, red dots denote the 20 most accessible sequences that are not crystal structures, while colored dots correspond to the crystal structures in Panel E, all falling into the same well, consistent with their conserved docking modes. For the synthetic interface, dots are colored according to the colors of the structures in Panel D based on their corresponding clusters. To visualize the geometry of each landscape, we chose a random ordering of the 20 most accessible wells, with the exception of the natural interface which always centers the deepest energy well, using a random ordering for the rest.

To visualize the relationships among the enriched sequences in the round 5 NGS data, we constructed an SSN (39). Sequence communities within the SSN were merged into single nodes, resulting in a community map comprising seven distinct clusters (Fig. 3A). Although the overall amino acid composition across all eleven library positions remained diverse, sequence logos for each cluster showed patterns of convergence, suggesting distinct coevolutionary pathways to the different clusters. Surprisingly, we only saw clusters with convergence of both interfaces, suggestive that the partners had ‘homed’ in on one another through their library patches, albeit with imperfect structural alignments of the libraries (discussed below) (Fig. 1C). To further illustrate the specificity and orthogonality of interactions, we used a Circos plot (40) to represent pairwise relationships between Z-A and Z-B proteins within the round 5 NGS data (Fig. 3B). The plot revealed minimal cross-reactivity (orthogonality) between clusters, with most sequences paired within their respective clusters.

To explore the structural characteristics of coevolved complexes, we selected a representative of the most enriched sequences from each cluster and validated their binding using the cleavage-capture assay, confirming strong binding (Fig. 3C). For six of the seven clusters, we solved crystal structures of one coevolved complex, with resolution ranging from 1.43 to 2.85 Å (Table S1). Structural analysis revealed considerable diversity in the docking orientations across the complexes, whilst maintaining unidirectional polarity (i.e., we did not see reverse docking orientations), analogous to the hands of a clock. Docking angles varied by up to 91.1° between A5B5 and A7B7 complexes, and none of the coevolved docking poises resembled the initial docked poly-Val model (Fig. 3D and fig. S4). This wide diversity in ratchet-like docking orientations contrasts with our previous study (27), where highly constrained docking modes were observed in coevolved complexes involving the natural interface of the Z-domain and its affibody binder (Fig. 3E). Inter-residue distances across the natural interface complexes were highly conserved, whereas those of the synthetic interface complexes varied substantially (fig. S5). The buried surface areas (BSA) across the coevolved interfaces ranged from 1080 to 1475 Å^2^, which are on the smaller side for PPIs but remain consistent with previous structural studies (11, 41). To assess interface packing, we calculated shape complementarity (sc) (42), packstat scores (43), and analyzed cold spots (44-46) across all solved natural and synthetic interface complexes (fig. S6). We identified a single cold spot (44) in the parent structure of the natural interface (fig. S6A), whereas no cold spots were detected in any of the coevolved natural or synthetic interfaces, indicating that the coevolved interfaces are well-packed.

Despite the library composition being limited to generally hydrophobic residues, the diversity in docking orientations of the synthetic interface clusters, spanning a range of approximately 90 degrees in 15 degree increments, suggests distinct fitness landscapes were sampled during coevolution. To elucidate characteristics of the overall fitness landscape, we employed a simple *in silico* coevolution simulation (one mutation at a time, and only improving the fitness), generating 10 million trajectories for each interface, using our learned fitness function from SPM (Methods). For each sequence, we computed its accessibility which is defined as the fraction of all trajectories that landed at that sequence. Highly accessible sequences represent energy wells in the energy landscape where coevolutionary pathways converged (energy is the negative fitness (47)). We noticed a striking difference in the number and depth of energy wells between the natural and synthetic interfaces (Fig. 3F). For the natural interface, the energy landscape was dominated by a single highly accessible well containing all strongly binding sequences. In contrast, the energy landscape of the synthetic interface contained a multiplicity of comparably accessible wells that each contained strongly binding sequences (Fig. 3F and fig. S7). These differences—namely difference in well depths and accessibilities between natural and synthetic—were substantiated as statistically significant through a bootstrap-based statistical test (fig. S8 and Method), with p-values respectively P= 4.7x10^−310^ and P=2.9x10^−147^. We additionally experimentally characterized the geometries of the energy landscapes as follows. We measured K_D_ values by SPR for a subset of the variants in the energy landscape for each of synthetic and natural interfaces. Specifically, we leveraged K_D_ measurements for 6 variants in the synthetic interface experiments appearing in the specificity matrix (fig. S9), that happen to be among the 20 most accessible appearing in our energy landscape plot. Their measured K_D_ values ranged from 0.4-8.8 μM, which corresponds to an energy gap of 1.8 kcal/mol. For the natural interface, we obtained SPR measurements for the variant corresponding to the deepest energy well and 4 variants that lie at the bottom of 4 other wells in the natural landscape. The K_D_ value of the variant corresponding to the deepest energy well was 2.4 nM (27) and for the other 4 sequences, ranged from 6.7 μM to >100 μM (i.e., beyond the SPR measurable limit of 100 μM) (fig. S9D), for which the smallest energy gap between the deepest well variant and the other variants is 4.7 kcal/mol (from the 6.7 μM variant). Importantly, this natural interface landscape energy gap (conservatively estimated because it is the smallest one) is 2.6 times larger than the measured energy gaps in the synthetic interface.

### Structural insights into specificity and cross-reactivity between clusters

**Figure 4.**
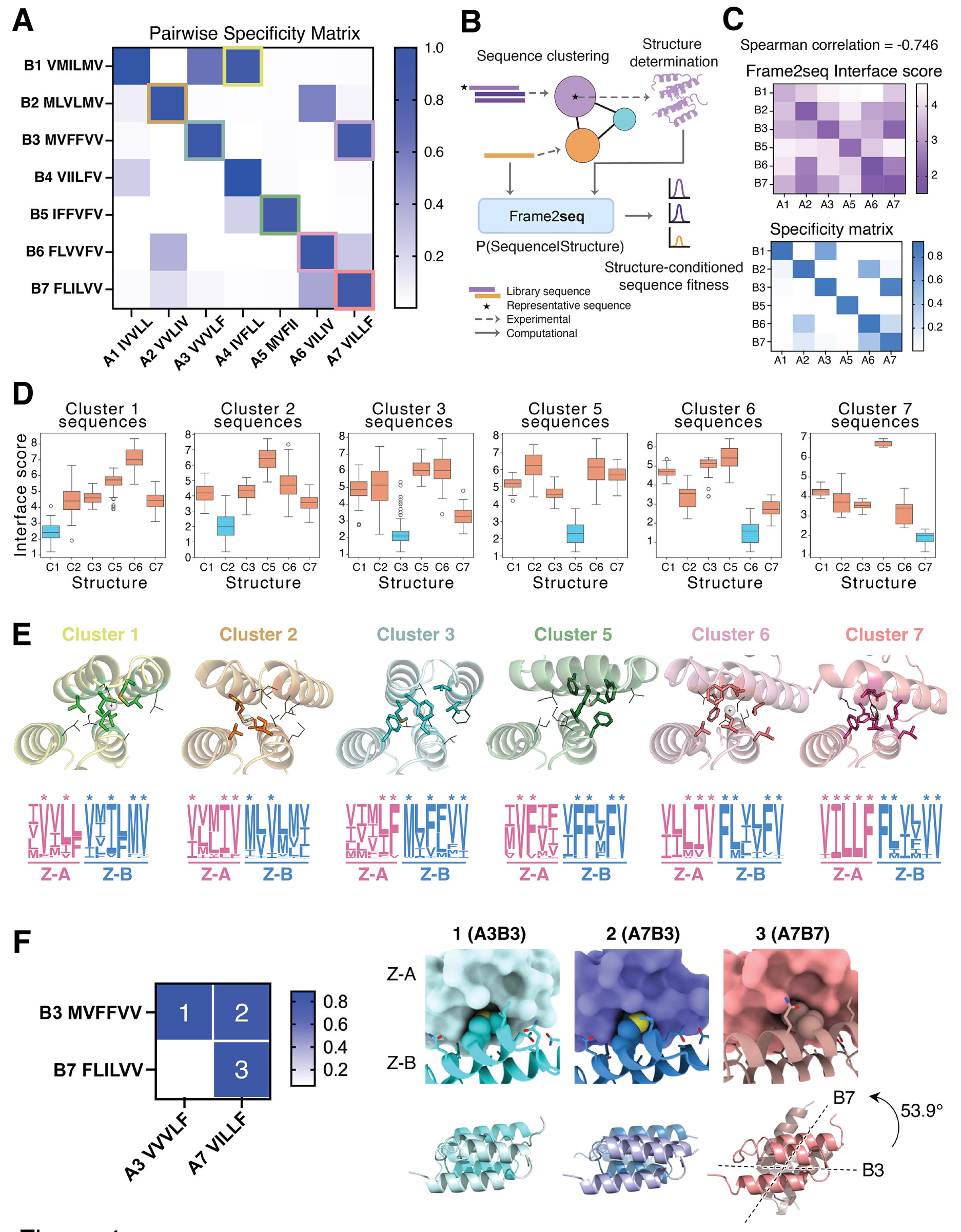
Structural insights into specificity and orthogonality between clusters. (A) Specificity matrix of all possible Z-A and Z-B combinations of representative pairs in Fig. 3C. Binding affinities measured by on-yeast cleavage-capture assay were normalized to the highest affinity (A4B4 complex) and non-interacting control (11xAla mutant). The matrix shows high intra-cluster specificity and some inter-cluster cross-reactivity (e.g., A4B1, A7B3). Data are mean of n = 3 independent replicates. (B) Workflow for scoring sequence-structure compatibility using Frame2seq. All library sequences are scored for compatibility with their representative structure. (C) Experimentally determined specificity matrix of representative sequences (top) and predicted Frame2seq interface score matrix of all library sequences (bottom) (Spearman correlation = −0.746). Frame2seq scores are negative log likelihoods (lower scores mean higher fitness). (D) Frame2seq interface scores of round 5 sequence similarity network (SSN) cluster sequences on all representative crystal structures (see Methods for details on representative structure selection). The correct (cognate) sequence-structure pairings are in blue, and the incorrect (noncognate) pairings are in orange. (E) Interface structures reveal converged residue interactions. (top) Interface structures of coevolved complexes highlight converged residues in stick representation, with other library residues shown as lines. The center of mass (COM) of the converged residues is depicted as a grey sphere at the interface, illustrating their clustering in proximity. Each structure exhibits distinct COM positions, reflecting different interaction and docking geometries across clusters. (Bottom) Sequence logos of each cluster illustrate residue convergence, with converged residues marked by asterisks. These data demonstrate how pairwise residue convergence underpins cluster-specific interactions and correlates with distinct docking orientations, highlighting the structural determinants of specificity within coevolved interfaces. (F) Pairwise specificity matrix highlighting biased cross-reactivity between cluster 3 and 7 pairs (left). A7 exhibits dual specificity by binding both B3 and B7, while A3 binds only B3. The interaction intensity in the left matrix is derived from the specificity matrix in Fig. 4A. Interfaces and top views of the A3B3 (pale cyan/cyan), A7B3 (slate/skyblue), and A7B7 (salmon/dark salmon) crystal structures (right). Z-A subunits are shown as surface representations and Z-B subunits as cartoons, with residue 29^B^ side chain atoms shown as spheres. Top views reveal distinct docking geometries: in the A7B7 complex, the Z-B subunit is rotated by 53.9° relative to the A7B3 complex. Models of putative A3B7, which fails to form a stable complex, are presented in fig. S13.

To further elucidate the specificity and cross-reactivity of coevolved complexes, we analyzed the structural and sequence features of representative pairs from each cluster. Specificity between the seven representative pairs was validated by measuring relative binding affinities of all possible combinations of Z-A and Z-B proteins (Fig. 4A). The resulting specificity matrix revealed strong intra-cluster specificity, with cognate pairs occupying the diagonal positions. Some instances of cross-reactivity were observed, including A4B1 and A7B3 mixed complexes, which formed between non-cognate pairs. That clusters can cross-react, despite evolving distinct sequence and structural solutions, suggests that the different docking modes are separated by shallow energetic barriers, consistent with the fitness analysis (48). To complement these analyses, we measured solution affinities (K_D_) by SPR for all combinations in the specificity matrix. Cognate pairs, as well as the noted cross-reactive pairs (A4B1 and A7B3), bound in the sub- to low-micromolar range (0.3 - 1.7 μM). Importantly, the solution K_D_ values correlated strongly with the on-yeast cleavage–capture HA-tag readout (Spearman r = 0.700, Pearson r = 0.868, R^2^ = 0.754; fig. S9), confirming that the specificity matrix accurately reflects binding in solution. We also examined whether biophysical parameters aligned with these affinities (11); however, common metrics such as buried surface area (BSA), shape complementarity (sc), and the hydrophobic fraction of buried surface area (% hydrophobic ΔSASA) showed no correlation with K_D_, underscoring that structural features alone are insufficient predictors of affinity in our synthetic interfaces (fig. S10).

The experimental cross-reactivity matrix for the 7 representative sequence pairs implies that a surprisingly high degree of orthogonality arises from the relatively limited choice of hydrophobic residues at each library position. To probe the generality of this observation beyond individual sequences, we sought to comprehensively map all sequences in the clusters to the six crystal structures. To do so, we used Frame2seq (32) to quantify sequence-structure compatibility in silico (Fig. 4B, Methods). We provided the experimentally-solved crystal structure backbones and sequences outside the library positions as inputs to the model and computed Frame2seq model scores to estimate sequence-structure compatibility for the library residues at the interface (Methods; Eq. S1). We analyzed (i) chain pairing specificity (Fig. 4C) and (ii) compatibility of all selected sequences in each cluster with each of the six crystal structures (Fig 4D). To assess chain pairing specificity, we scored all possible Z-A and Z-B combinations with cognate (diagonal) and non-cognate (off-diagonal) chain pairings (Fig. 4C). The resulting chain pairing confusion matrix shows a strong correlation to the experimentally validated specificity matrix (Spearman correlation = −0.746). This level of predictive accuracy of Frame2seq for specificity prediction is surprising given the relatively shallow fitness landscapes between clusters and is encouraging for using such models for mapping sequence-structure space. To further assess the generalizability of Frame2seq, we benchmarked it against the Flex ddG dataset of experimentally measured interface mutations (49), where the model successfully distinguished destabilizing from stabilizing mutations (fig. S11), consistent with its predictive accuracy on our synthetic complexes. To assess sequence-structure mapping, we scored all possible paired cluster sequences for compatibility with each of the six experimentally-solved structures representing these clusters (Fig 4D). Cognate sequence – structure pairings are consistently scored by Frame2seq to form more favorable (lower scoring) interfaces. These results suggest that the sequences in each of the identified clusters indeed map well to the six representative crystal structures.

We examined the high-resolution crystal structures of coevolved complexes from each cluster to understand the basis of both specificity and cross-reactivity. The structures revealed a wide range of interface packing solutions, with converged residue interactions driving specificity within clusters (Fig. 4E). Converged residues, marked with asterisks on the sequence logos, were highlighted in stick representation on the structures. These residues cluster closely within the interfaces, emphasizing that pairwise convergence observed in NGS data correlates strongly with cluster-specific interactions and distinct structural determinants that define docking orientations.

Interestingly, beyond intra-cluster specificity, we also observe inter-cluster cross-reactivity that can result in dual or multi-specificity by a single binding partner. To assess how common these cross-reactivities are, we quantified inter-cluster cross-reactivity among the sequences from the SSN clusters (Fig. 3A). We found that 19.9% of Z-A sequences exhibited at least one inter-cluster cross-reactivity, whereas only 3.4% of Z-B sequences did so, indicating that cross-reactivity occurs primarily on the A chain (fig. S12). This is exemplified by the A7 protein, which can bind strongly with both B3 and B7 with comparable affinities (K_D_= 487 nM for A7B7; 551 nM for A7B3) (Fig. 4F and fig. S9). Structural analysis revealed that A7 adopts different docking modes depending on its partner. In the A7B3 complex, the Z-B subunit is rotated by 53.9° relative to the A7B7 complex, and the H1 helix of A7 shifts by 4.1 Å (Fig. 4F and fig. S13). This demonstrates how sequence convergence within clusters supports intra-cluster specificity, while inter-cluster cross-reactivity emerges from docking plasticity, allowing a shared partner to navigate distinct energy wells through structural adaptation. Notably, cross-reactivity was also directional, with A7B3 forming but another possible cross-reactive pair A3B7 failing to interact, due to a steric clash caused by a Met29^B^Phe substitution in Z-B (fig. S13). Interestingly, position 29^B^ strongly converges to Met in cluster 3 and Phe in cluster 7 (Fig. 4E), suggesting that these residues contribute to cluster-specific docking preferences and impose structural constraints on inter-cluster cross-reactivity.

### Structural and epistatic signatures underlying synthetic and natural protein-protein interfaces

**Figure 5.**
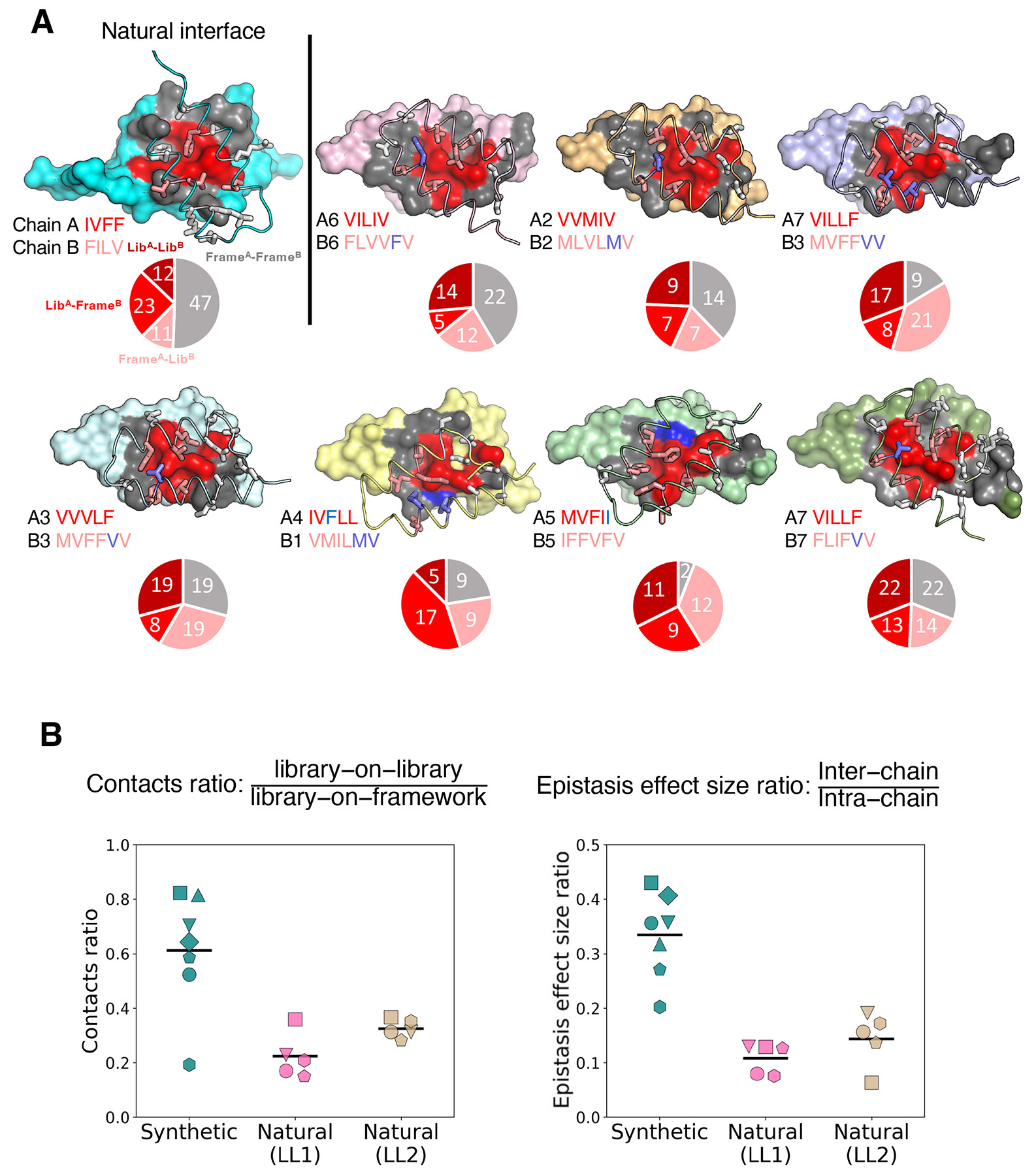
Structural parsing of interface composition and analysis of epistasis. (A) Contacts between Z-domain chains. For each structure, chain A is shown as a surface representation and chain B as a cartoon with contacting residue side chains shown as sticks. Noncontacting residues are colored as in panel A, noncontacting library residues are colored blue or slate, contacting framework residues are gray or white, chain A contacting library residues are red, and chain B contacting library residues are salmon. The noncontacting helix of chain B is omitted for clarity. Below each structure is a pie chart of atomic contacts between helical residues: Framework-framework (gray), A library – B framework (red), A framework – B library (salmon), A library – B library (dark red). A representative natural interface Z-domain-affibody pair (LL2.c22) is shown in cyan. (B) Dot plots of contact ratios (library-on-library contacts over library-on-framework contacts) and SPM epistasis effect size ratios (inter-chain epistasis effect sizes over intra-chain epistasis effect sizes) for the crystal structures. For each type of interface, the black horizontal bar marks the average contacts ratio or epistasis effect size ratio of the corresponding crystal structures. Each crystal structure uses the same markers in the two plots.

Our hypothesis in the design of the coevolution experiment was that the library patches would converge on one another through inter-chain epistasis. To ask how structural convergence occurred, we first curated all atomic contacts < 4 Å between subunits in all of our crystal structures (Fig. 5A, Table S2). We then classified randomized positions as “library” and constant positions as “framework”, denoting contacts as library-on-library ‘LL’(A_lib_-B_lib_), library-on-framework ‘LF’ (A_lib_-B_frame_, A_frame_-B_lib_), or framework-on-framework ‘FF’ (A_frame_-B_frame_). The cores of all interfaces are dominated by LL contacts, which presumably act as “hotspots” to initiate complex formation at the early stages of selection. The centrally located LL patches are not perfectly structurally coincident, thus LF contacts occur at the peripheries of the misaligned LL regions, and FF interfaces occur adventitiously at the outermost edges of the interfaces.

We sought to quantify the distinct patterns of structural interactions in the natural versus synthetic interfaces by comparing their contact ratios, defined as the number of LL contacts (A_lib_-B_lib_) divided by the number of LF contacts (A_lib_-B_frame_ and A_frame_-B_lib_) in the crystal structures. Synthetic interfaces spanned a wide range of contact ratios, with an average of 0.61; whereas the natural interfaces had a narrower range of contact ratios and a lower average: 0.22 for the LL1 library and 0.33 for the LL2 library (Fig. 5B left). We hypothesized that the library-to-library interactions might be related to inter-chain epistasis, whereas library-on-framework interactions might be related to intra-chain epistasis. Indeed, we found that a higher ratio of inter-chain over intra-chain epistasis effect sizes tended to correspond to a higher ratio of LL to LF contacts (Fig. 5B right, fig. S14). Notably, the *de novo* complex formation has a higher proportion of LL contacts compared to the natural complex, suggesting LL contacts may be primary drivers of *de novo* complex formation. Presumably this difference arises from the natural protein partners being trapped in the deep energy well of the natural docking mode; within that well, the primary role of epistasis is to optimize contacts in the existing interface rather than to explore new interfaces.

### Epistatic dissection of coevolution in synthetic and natural interfaces

We next sought a more detailed understanding of how epistasis and structure play out during coevolution. Physical interactions could form in part through intermolecular (ZA-ZB) epistasis of library amino acids on their respective surfaces, and also through intramolecular epistasis within each binding partner (ZA or ZB). Intramolecular epistasis could preorganize a binding surface independently of a partner to make it more likely to bind to any partner. To determine which mechanisms may drive different stages of coevolution, we leveraged our epistasis analysis to reveal critical interactions in the overall SPM-predicted fitness landscape as well as those in the individual SPM-predicted fitness landscapes associated with each selection round (see Methods, “Extraction of epistasis importance from SPM-predicted fitness landscape” section). We also applied a similar analysis to the Frame2Seq sequence-structure compatibility scores.

**Figure 6.**
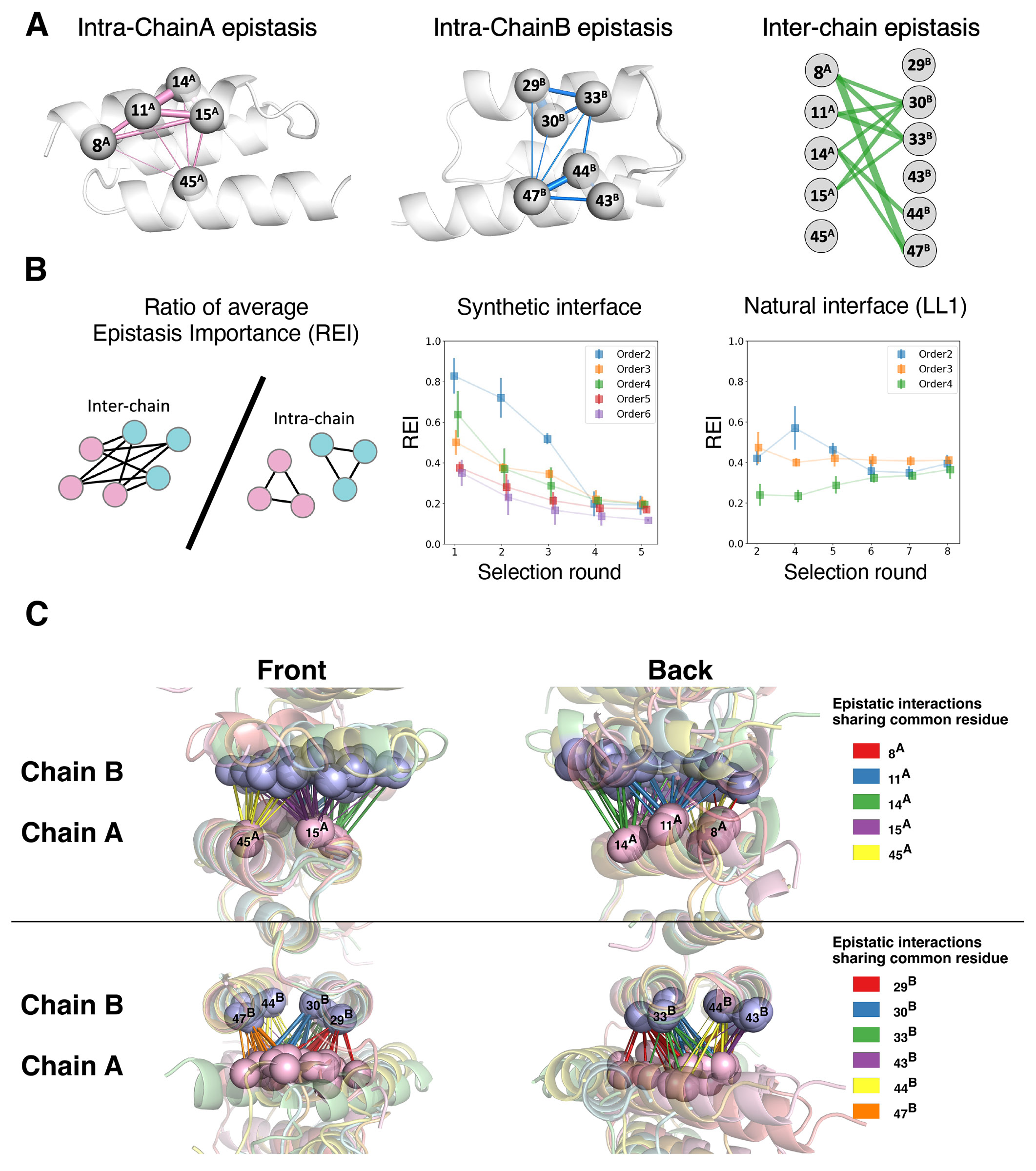
Epistatic contributions between coevolved pairs. (A) Left and center: Visualization of intra-chain pairwise Selection Probabilistic Modeling (SPM) epistasis overlaid onto the crystal structure of A5B5. Widths of the bonds connecting the library residues are proportional to the strength of the corresponding epistasis importance. Right: Top 10 inter-chain pairwise SPM epistasis importance. The widths of the lines are proportional to the corresponding epistasis importance. (B) Left: Illustration of Ratio of average Epistasis Importance (REI). It is computed as the ratio of the average inter-chain SPM epistasis importance per term over the average intra-chain SPM epistasis importance per term for each order of interaction. Middle and right: Comparison of REI for each round of selection experiments of synthetic and natural interfaces. (C) Pairwise epistasis terms per cluster structure, overlayed and aligned on Z-A (above) and Z-B (below) when viewed from the front (left) and back (right) of the complex. Spheres represent library residues, and bars represent epistatic interactions where thickness represents epistasis strength as computed by Frame2seq and color represents the residue shared amongst all such interactions (which is also depicted on the legend in the right).

First, leveraging the SPM fitness, we found a strong correlation between pairwise epistasis importance and physical proximity (Spearman correlation=-0.550, fig. S15). Epistasis “importance” refers to the maximum amount that a given interaction can change the fitness. The weaker correlation for inter-chain pairs likely arises from diverse docking modes in the crystal structures, as some pairwise epistasis may be important for a particular docking mode. Residues involved in the strongest pairwise intra-chain epistatic interactions tended to lie on the same helix at the binding interface (Fig. 6A).

Next we inspected how contributions of intra-chain and inter-chain interactions likely changed over selection rounds by leveraging our round-specific epistasis analysis as a proxy. As expected, we found that both intra- and inter-chain epistasis increased with increasing selection strength (*i.e.*, increasing round number) (fig. S16). The relative contribution of inter-chain over intra-chain epistasis importance decreases with increasing selection strength (Fig. 6B), suggesting that after establishing binding contact in the early rounds, each chain may then individually focus more on fine-tuning of its own energetics. In contrast, when performing the same analysis on the original Z-domain-affibody interface, the relative contributions did not systematically decrease (Fig. 6B), presumably because binding contact did not need to be established in the first place.

We additionally performed epistasis analysis on the sequence-structure compatibility scores derived from Frame2seq using each of the six crystal structures (Methods, “Extraction of structure-conditioned epistasis importance using Frame2seq” section). We found that many of the inferred epistatic interactions were conserved (involving equivalent residues) across all six structures, despite the different docking modes (Fig. 6C). Furthermore, several of the identified interactions were also seen in the SPM global fitness epistasis analysis (fig. S17). These results suggest that a set of key interactions are important in the formation of the interface, and that the different docking modes “pivot” around these core interactions.

### Seed sequences in the coevolutionary paths

We wondered whether there were primordial “seed” contacts that could serve as the earliest sentinels of protein binding emerging from the formerly silent surfaces in the selections. Moreover, such key interactions could be preconditions that favor an evolutionary path toward a specific binding conformation. To attempt to extract the identity of seed sequences, we analyzed our simulated coevolutionary trajectories and curated potential seed sequences as those that are weak binders (with SPM-predicted fitness between 18.35 and 22.07) with relatively high “exclusivity” and “contribution” towards their most likely energy well (the well that their trajectories fall into most frequently; we considered only wells that correspond to sequences in Fig. 4A whose binding affinity had been experimentally measured with normalized HA-tag binding affinity > 0.8). Exclusivity of a sequence for a well refers to the fraction of trajectories from that sequence that end in that well; contribution of a sequence to a well is defined as the fraction of all trajectories ending in that well that are accounted for by that sequence (Methods).

**Figure 7.**
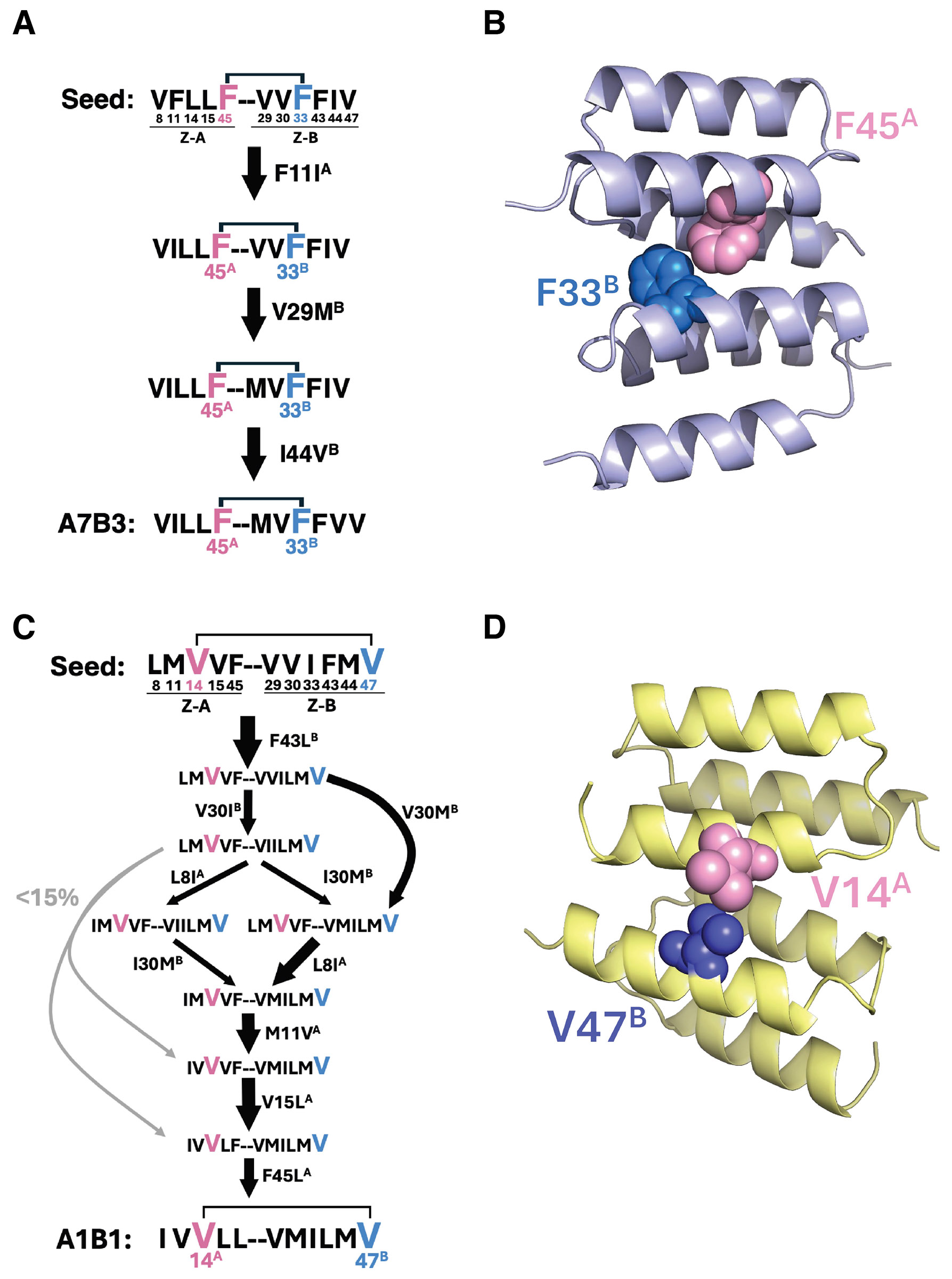
Seed sequences initiate coevolutionary trajectories. (A) Illustration of the only energetically favorable coevolutionary path from the identified seed (VFLLF--VVFFIV) to A7B3. The conservation of the top-ranking inter-chain epistasis between F45^A^-F33^B^ is highlighted. (B) Visualization of the conserved top ranking inter-chain epistasis F45^A^-F33^B^ on the crystal structure. (C) Illustration of energetically favorable coevolutionary paths from the identified seed (LMVVF--VVIFMV) to A1B1. The widths of the arrows are proportional to the number of simulated trajectories visiting the corresponding paths. Gray arrows represent minor paths that were collectively visited less than 15% of all the simulated coevolutionary trajectories. Conserved top ranking pairwise inter-chain epistasis V14^A^-V47^B^ is highlighted in each sequence in the paths. (D) Conservation of top-ranking inter-chain epistasis visualized on the structural model of A1B1: V14^A^-V47^B^ is the highest or the second highest ranking inter-chain epistasis effect size for the seed, all the waypoints and A1B1. The structural model of A1B1 is built by making the F14^A^V point mutation of the crystal structure A4B

This curation yielded plausible seed sequences for sequences A7B3 and A1B1. From these seed sequences, we selected one sequence with high exclusivity and relatively high contribution for each of A7B3 and A1B1 for further analysis (fig. S18). We found only one energetically favorable coevolutionary path to A7B3 from its selected seed (Fig. 7A and fig. S19). Our epistasis analysis identified F45^A^-F33^B^ as the highest-ranking inter-chain pairwise epistasis effect size along this path, suggesting its pivotal role in progression from weak to strong binding partners. This interaction is physically located in the core of the protein interface (Fig. 7B). In contrast to the case of A7B3, for A1B1 we found multiple viable coevolutionary paths from the selected seed (Fig 7C and fig. S18B). For all of the sequences along these paths, the interaction V14^A^-V47^B^ ranked as the highest or second highest in inter-chain pairwise epistasis effect size, suggesting its pivotal role in binding evolution; residues in this interaction pair are in close physical contact with each other (Fig. 7D). Altogether, these results suggest that key interactions not only help to usher the evolutionary path toward a specific binding conformation from a seed but are also maintained along the path. Moreover, distinct key interactions may drive the differences between docking modes.

## Discussion

We have used a coevolution platform to try to understand the differences between surfaces of proteins that have evolved to bind to other proteins, versus those that have not. Our strategy was to simulate the ontogeny of new protein-protein interactions *in vitro* between “silent” surfaces of proteins with no natural history of protein-protein interactions and then deconstruct this process. By integrating experimental coevolution and statistical machine learning, this platform has revealed how structural and epistatic principles, including “seed” interactions, shape binding specificity and adaptability. Synthetic complexes adopted a range of docking modes, distinguished by subtly different interfaces, by sampling shallow, sawtooth energetic landscapes. This was not seen during coevolution of a complex mediated by natural Z-domain binding surfaces, that appeared to reside within a deep energy well (27).

Our results offer insight into why surfaces of proteins that have not evolved to engage ligands are notoriously difficult to drug (50). Natural protein binding sites have been evolutionarily refined over millennia, appearing to possess structural and energetic imprints that make them conducive for binding and as drug targets (6, 51). It is well established that combinatorial libraries undergoing unbiased selections, such as peptide and antibody phage libraries, frequently converge on the natural binding sites of proteins, which serve as the most “druggable” sites for both small molecules and protein therapeutics (6). In contrast, regions of protein surfaces that do not engage ligands were likely evolutionarily “counter-selected” to avoid spurious interactions with non-specific proteins, and this likely contributes to lack of “druggability.” Targeting non-binding regions of proteins may be limited by a relative lack of evolutionary fitness that results in a dearth of structural features conducive to binding (1, 2, 6). Consequently, non-binding surfaces of proteins may be constrained to shallow energetic landscapes that have evolved to avoid non-specific interactions with irrelevant molecules. Our synthetic coevolution experiment overcame this limitation through molecular diversity, but even so, the resulting complexes exhibited a sawtooth energetic landscape that explored parallel evolutionary pathways to diverse structural solutions. This plasticity may have been further facilitated by the hydrophobic nature of our designed library, which promotes chemically compatible packing across multiple configurations, as shown by the lack of cold spots in the synthetic interfaces despite the shallow energy landscapes (38, 44). Underscoring this shallow energetic landscape, we observe that docking modes can interconvert in a ratchet-like fashion through only a few amino acid mutations, illustrating a remarkable potential for plasticity in molecular recognition.

A limitation of our coevolution experiment is that we used a biased library restricted to hydrophobic amino acids. We designed our library to be within the experimental limits of diversity of yeast display, which is about 10^9^. We chose 11 sites to cover a surface area that lead to binding interfaces on the smaller side for PPI (~1080-1475 Å^2^) but still common (11, 41), and allowed complete sampling of the theoretical diversity of the library (3.6×10^8^). We also wanted the library to be similar in composition to our previous library we used for remodeling the protein Z-affibody interface (27) so that we could make comparisons to the synthetic interfaces in the current manuscript. Indeed, several of our results are consistent with previous studies on evolution of PPI based on analysis of all 20 natural amino acids. For example, we found that dual-binding states (i.e., promiscuous intermediates (13)) frequently occur along shortest viable mutational paths between co-evolved crystal backbones (as per our SPM-estimated fitness); this result suggests that the biased libraries are capable of mimicking characteristics of natural repertoires at a systems level. We also did not see correlations between measured biophysical parameters (buried surface area (BSA), shape complementarity (Sc), and the hydrophobic fraction of buried surface area [% hydrophobic ΔSASA]) and pK_D_ as measured by SPR for our synthetic interfaces, as previously shown in (11). Consistent with well packed natural PPI using all twenty amino acids, we found that the synthetic interfaces were well packed, lacking cold spots (44, 45), showing that even our limited library is capable of evolving natural-like interfaces.

Concordant with previous studies showing the importance of epistasis in natural protein evolution (15, 46, 52), we also found that epistasis plays a critical role in the evolution of both synthetic and natural interfaces, but distinct patterns emerged in each. Synthetic interfaces exhibit higher inter/intra epistasis importance ratios during early selection rounds, suggesting that inter-chain interactions played a stronger role in determining binding modes at the onset of selection, compared to the affinity maturation stage (Fig. 5B). These inter-chain interactions may have provided an initial structural foothold for binding, enabling intra-chain refinements in later rounds to optimize stability and specificity. We speculate that the original high affinity Z-domain-affibody complex—trapped in a deep energy well that constrains its ability to adapt to the epistasis through reorientation of docking modes—distributes the epistasis more evenly for interface repacking. As a result, natural interfaces maintain a balanced inter/intra epistasis importance ratio throughout selection.

It is instructive to ask how our results on a model system inform our understanding of the initiation step of complex formation during natural protein-protein coevolution. Presumably, naïve proteins acquired mutual affinity through evolution of mutations on complementary surfaces under a selective pressure over a long time period (53). In such a setting, natural coevolution is iterative over time, where advantageous mutations that stabilize the complex accumulate and are fixed, followed by further sampling of mutants until a functional threshold, such as affinity, is met, at which point further affinity-enhancing mutations become superfluous (29, 54, 55). In our system, seed interactions, such as F45^A^-F33^B^ in A7B3 and V14^A^-V47^B^ in A1B1, appear to act as stabilizing anchors, linking seed sequences to refined binding interfaces, while preserving evolutionary flexibility, as evidenced by the divergent coevolutionary trajectories. We suggest that such mechanisms likely model mechanisms of protein-protein evolution that occur in nature. During natural protein-protein evolution, it is unlikely that a suitable constellation of mutations would arise simultaneously at the seed stage that would confer high affinity in a single step (56). Thus, we suggest that our model system has captured the essence of a natural mechanism for the initiation of protein-protein complexes.

There are examples of artificially designed protein-protein interfaces that leverage molecular docking followed by side chain design calculations to create novel interfaces (57-59). These methods sample shape complementarity extensively through docking but generally lack the ability to capture nuanced side chain-guided interfacial plasticity because of the hierarchical nature of the design procedures, as well as challenges associated with overcoming local energy minima. Epistatic clusters of residues are secondary considerations to these types of design methods, typically relying upon the design algorithm to capture them. We used docked poses without specific sequences to set up the residue positions that undergo sequence drifts and came to sequences that convey orthogonality through new binding orientations, not easily designable by conventional methods (60, 61).

Artificial intelligence is making rapid strides in prediction and design of binding proteins (62-65), but these *in silico* approaches remain largely agnostic to the biophysical chemistry and evolutionary mechanisms by which protein-protein interactions (PPIs) are formed. Generative AI approaches are effective at producing binders to proteins, but are generally most effective targeting natural binding surfaces that have evolved to bind ligands (59). Both targeting naïve surfaces of proteins and achieving fine specificity among highly similar surfaces remain challenging. Understanding the structural principles underlying protein interfaces at a systems level, using large datasets of paired coevolved protein-protein complexes, could help to obtain deeper insight into molecular recognition and also bridge the gaps in current AI-based protein design approaches. Such challenges are difficult for existing models trained largely on static and monomeric structures, which do not explicitly capture how sequence changes reshape interfaces to mediate specificity and cross-reactivity. Our approach might provide insights to construct improved ML-based protein engineering strategies for “undruggable” therapeutic targets (41, 66-68).

## Materials and Methods

### Z domain-Z domain docking

A Z-domain was isolated from the crystal structure of Protein Z in complex with an in vitro selected affibody (PDB: 1LP1). The Z-domain structure was minimized using the the Rosetta FastRelax protocol, applying backbone coordinate constraints to preserve the overall fold. A poly-valine variant of the Z-domain, where all amino acids were substituted with valine, was generated using RosettaRemodel. This poly-valine Z-domain was then docked against a second poly-valine Z-domain using PatchDock. Docked models were ranked based on their approximate interface are. Models with an interface area greater than 1000Å^2^ were subjected to refinement with the Rosetta FastRelax protocol under the same constraints. The refined models were ranked by Rosetta Energy Units (REU), and the 25 lowest energy structures were manually inspected in Pymol. The final model was selected based on both energy and interaction mode preference (helices13 interacting with helices23) from the PatchDock set with interface areas excessing 1000Å^2^.

The original protein sequence was restored onto each Z-domain in the selected docked model, which underwent further refinement with Rosetta FastRelax. Target residues on the interface of one Z-domain were identified based off of this refined model. These target residues were used to generate a rotamer interaction field (RIF) with RIFDock. A second round of PatchDock was performed, targeting the same residues used during the RIF generation step. PatchDock models with an interface area greater than 1000Å^2^ were used as seeds for RIFDock runs. RIFDock-generated models underwent additional refinement using the FastRelax protocol with coordinate constraints and were ranked by energy. The lowest energy models were visually inspected in Pymol, revealing a convergence to a docked conformation within 0.45Å of the best PatchDock model. This converged model was selected for further sequence design.

The docked model underwent refinement using RosettaDock local-refinement as well as side-chain packing and interface redesign with Rosetta FastDesign. Models were filtered by ddg (threshold = −15) and SASA (threshold=800). Redesigned models were ranked by energy, and top-ranking designs were analyzed to identify trends in residue preferences, which guided the selection of positions for constructing a degenerate codon library.

### Protein expression and purification

The protein Z domain-encoding DNA plasmids were inserted into the pET28 bacterial expression vector. The vector includes the Z-domain gene with either a C-terminal His_6_-tag or biotin-acceptor peptide tag (BAP tag, GLNDIFEAQKIEW) followed by His_6_-tag, inserted between the *NcoI* and *XhoI* sites of pET28b vector (Novagen). For expression, the plasmids were transformed into E. coli BL21 (DE3) cells, which were cultured in TB medium containing 50 mg/L kanamycin at 37°C. When the optical density at 600 nm (OD_600_) reached 0.6, protein expression was induced with 0.5 mM isopropyl-β-D-thiogalactoside (IPTG), and the cultures were incubated overnight at 25°C before cell harvesting. Protein purification was carried out using Ni^2+^-NTA agarose column chromatography (Ni-NTA, Qiagen), followed by further purification with size-exclusion chromatography on a Superdex S75 10/300GL Increase column (GE Healthcare). The final protein products were stored in HEPES-buffered saline (HBS; 20 mM HEPES, pH 7.5, 150 mM sodium chloride).

### Yeast display of single-chain Z domain dimers

Single-chain Z protein dimers were expressed on the surface of *Saccharomyces cerevisiae* strain EBY100 (Invitrogen, cat. no. C839-00) through fusion to the C-terminus of the Aga2 protein. The dimers, linked via a GS-linker containing a 3C protease cleavage site, were positioned between an N-terminal cMyc epitope and a C-terminal HA tag. The construct, formatted as N-cMyc-ZA-linker-ZB-HA-C, was cloned into the pCT302 vector (Addgene #41845). Competent yeast cells were transformed with Z-domain plasmids using a yeast transformation kit (Zymo Research T2001) and plated on SDCAA agar plates (Teknova). Plates were incubated at 30°C for two days until colonies formed. Single colonies were picked and cultured in SDCAA media (pH 4.5, 20 g dextrose, 6.7 g yeast nitrogen base, 5 g bactocasamino acids, 10.4 g sodium citrate and 6.4 g citric acid monohydrate dissolved in 1 liter of deionized H_2_O, supplemented with 10 ml of Gibco^™^ Penicinillin-Stereptomycin, 10,000 U/ml) until reaching an OD600 of 10. Cultures were then induced at 20°C for 24 hours by diluting to an OD600 of 1.0 in SGCAA medium (similar to SDCAA but containing 20 g galactose instead of dextrose). Protein display levels were validated by staining cells with Alexa Fluor 647-labeled anti-HA antibody (1:50 dilution; Cell Signaling Technology, cat. no. 3444S). Fluorescence signals were analyzed using flow cytometry (Beckman Coulter, CytoFLEX).

### On-yeast cleavage-capture assay

For the single clone cleavage-capture assay, colonies were selected from transformed EBY100 cells that were plated on SDCAA agar plates. A total of 5 × 10^5^ induced yeast cells were stained with Alexa Fluor 647-labeled anti-HA antibody at a 1:50 dilution. After staining, the cells were washed thoroughly with MACS buffer (autoMACS^®^ Running Buffer, Miltenyi, cat. no. 130-091-221) to remove unbound antibodies. The washed cells were then incubated in 20 μL of 3C protease cleavage solution, prepared by diluting lab-made 3C protease to 0.4 mg/mL in MACS buffer, and maintained at 4°C. At each time point, 2 μL of the sample was taken and diluted in 100 μL of ice-cold MACS buffer. The fluorescence intensity of the samples was measured using flow cytometry. To evaluate the affinity between two interacting proteins, the mean fluorescence intensity (MFI) at each time point was normalized to the MFI before cleavage, expressed as a percentage of the maximum MFI.

### Yeast displayed libraries

A site-directed mutagenesis library was generated through assembly PCR using DNA sequences containing degenerate codons. The gel-purified PCR product was combined with a linearized pCT302 vector and introduced into EBY100 cells via electroporation (69). Following electroporation, the cells were incubated in YPD medium at 30°C for one hour before being transferred to SDCAA media. To assess transformation efficiency, serial dilutions of the recovered cells were plated onto SDCAA agar plates. After a 2-day incubation, protein expression was induced in SGCAA media as described above.

Library DNA sequences are listed below.

GTTGATAATAAATTTAATGCA**DTS**CAATGG**DTS**GCATTT**DTSDTS**ATTTTGCATCTGCCCAATTTGAACGAGGAACAGAGAAACGCTTTCATACAGTCTCTAAAAGATGATCCAAGTCAATCAGCAAATTTA**DTS**GCCGAAGCTGCGGCCTTAAATGCCGCTCAAGCGCCTAAGGAATTCGGCGGAGGTGGGAGSCTGGAAGTTCTGTTCCAGGGTCCGGGAGGCGGCGGGAGCGGATCCGTTGACAACAAGTTTAACAAAGAGCAGCAAAATGCGTTTTACGAGATATTACATCTTCCGAATCTTAATGAGATACAGAGGAAT**DTSDTS**ATTCAG**DTS**CTGAAAGATGACCCTAGCCAGAGCGCC**DTSDTS**CTGGCT**DTS**GCGAAGATCGCAAACGATGCACAAGCACCTAAA

Theoretical nucleotide diversity: 3.63 × 10^8^

Functional library size: 2.50 × 10^9^

### Selection of yeast-display libraries

Yeast-display library was enriched for interacting pairs through a combination of magnetic-activated cell sorting (MACS) and fluorescence-activated cell sorting (FACS), as previously described (27). Initial negative selection was performed using 10 times the theoretical diversity of the library to remove uncleavable variants caused by linker mutations. Positive and negative selections were alternated to enrich interacting pairs while minimizing the accumulation of uncleavable mutants. Library selection involved four rounds of positive selection using MACS (R1-R4) followed by one round of FACS sorting (R5) to achieve higher purity of interacting pairs. The specific methods for MACS and FACS selections were performed as described previously (27).

### Deep sequencing of yeast libraries

DNA was extracted from 5-10 × 10^7^ yeast cells per selection round using the Zymoprep II kit (Zymo Research). Unique 6-mer barcodes and random 8-mer sequences were incorporated into the flanking regions of the sequencing product through 30 cycles of PCR amplification. The amplified region covered library positions for both Z-A and Z-B. A second PCR step was performed to add Illumina primer sequences, resulting in final products containing the format: Illumina P5-barcode-N8-read-Illumina P7. The final PCR products were purified using agarose gel electrophoresis, quantified with a Nanodrop, and subjected to deep sequencing on an Illumina MiSeq platform with a 2×300 V3 kit.

The amplicons were amplified using the following primers:

Illumina forward primer:

5'-AATGATACGGCGACCACCGAGATCTACACTCTTTCCCTACACGACGCTCTTCCGA-3'

Illumina reverse primer:

5'-CAAGCAGAAGACGGCATACGAGATCGGTCTCGGCATTCCTGCTGAACCGCTCTTC-3'

### Sequence library filter

To identify enriched oligopeptide pairs, a one-sided hypergeometric test was performed using the scipy.stats library. Counts of individual oligopeptides (A and B) and their pairwise combinations (AB) were extracted from the sequencing data. The total population size (M), counts of each A or B oligopeptide (n and N), and observed AB pair counts (k) were used as input parameters for the hypergeometric distribution. The survival function (P(X>(k−1))) was computed to determine p-values for each pair. Pairs with p-values below 0.05 and counts greater than or equal to 20 were retained as enriched sequences in the round 5 NGS data. The filtered results were stored for further analysis, ensuring that only statistically significant and highly abundant pairs were included in downstream analyses, such as sequence similarity networks and Circos plots.

### Sequence similarity network and Circos Plot

Coevolution data were imported from a CSV file containing filtered round 5 NGS data, where p-values were < 0.05 and read counts were ≥20, resulting in 862 enriched sequences out of a total of 416,872 unique round 5 sequences, which were used for plotting sequence similarity networks and a Circos plot. Sequence similarity networks and community maps were constructed using the igraph software package (70). Nodes in the edit distance-based networks represented unique Z-A/Z-B pairs, and edges were added between nodes when the edit distance between two pairs fell below a predefined threshold.

A Circos plot was generated using the PyCirclize library to visualize coevolutionary interactions among protein sequences. A constant score value was assigned for uniform visualization, and interaction categories were color-coded (e.g., red, orange, yellow) to differentiate cluster groups. The final Circos plot provided an effective visualization of complex coevolutionary networks, highlighting sequence clusters and interaction patterns.

### X-ray Crystallography

Some protein complexes were reductively methylated (71), as noted below, and all were digested with carboxypeptidases A and B, purified by size-exclusion chromatography, and concentrated. All crystals were grown by vapor diffusion using either 100 nl protein with 100 nl well solution or 100 nl protein with 80 nl well solution and 20 nl microseeds, and flash cooled with liquid nitrogen after cryoprotection as noted below.

Methylated A4B1 was concentrated to 245 mg/ml and crystallized against 0.1 M bis-tris-propane pH 9.0 and 30% polyethylene glycol (PEG) 6000. Crystals were cryoprotected by drawing through a drop of paratone N. Diffraction images were collected at the Advanced Light Source (ALS) beamline 8.2.1.

Initial crystals of A2B2 grew at 193 mg/ml from 0.1 M bis-tris pH 5.5 and 25% PEG 3350. These were used to make microseed stock to grow the final crystals at 228 mg/ml from 0.1 M bis-tris-propane pH 9 and 30% PEG 6000. Crystals were cryoprotected with addition of 30% glycerol. Diffraction images were collected at the Stanford Synchrotron Radiation Laboratory (SSRL) beamline 12-1.

Initial crystals of A3B3 were grown at 200 mg/ml from 0.1 M bis-tris pH 6.5 and 25% PEG 3350. A microseed stock from these crystals was used to grow the final crystals from 0.2 M NaCl, 0.1 M bis-tris pH 6.5, and 25% PEG 3350. Crystals were cryoprotected by drawing through a drop of paratone N, and diffraction images were collected at ALS beamline 8.2.1.

Crystals of A5B5 were grown with two cycles of microseeding. Initial seed crystals grew at 210 mg/ml from 0.2 M Li_2_(SO_4_), 0.1 M Tris pH 8.5, and 30% PEG 4000. Secondary seeds were grown with microseeding from 0.1 M HEPES pH 7.5 and 20% PEG 10,000. Final crystals were grown from 0.15 M DL-Malic acid pH 7.0 and 20% PEG 3350. Crystals were cryoprotected by drawing through a drop of paratone N, and diffraction images were collected at ALS beamline 5.0.1.

Methylated A6B6 was concentrated to 168 mg/ml and crystallized from 0.01 M CoCl_2_, 0.1 M MES pH 6.5, and 1.8 M (NH_4_)_2_SO_4_. Crystals were cryoprotected by drawing through a drop of paratone N, and diffraction images were collected at ALS beamline 2.0.1.

Initial crystals of A7B7 grown at 138 mg/ml from 0.1 M sodium cacodylate pH 6.5 and 1.4 M sodium acetate were used to prepare a microseed stock. Final crystals were grown at 192 mg/ml with microseeding from 0.1 M bis-tris pH 6.1 and 2.1 M (NH_4_)_2_SO_4_. Crystals were cryoprotected with addition of 3 M sodium malonate pH 6.0, and diffraction images were collected at the National Synchrotron Light Source (NSLS-2) AMX beamline.

Initial seed crystals of methylated A7B3 were grown at 167 mg/ml from 0.1 M Tris pH 8.5 and 25% PEG 3350. Final crystals were grown with microseeding at 136 mg/ml from 0.05 M MgCl_2_, 0.1 M HEPES pH 7.5, and 30% PEG monomethyl ether 550. Crystals were harvested without further cryoprotection and diffraction images were collected at SSRL beamline 12-2.

Diffraction data wee indexed, integrated, and scaled using either XDS (72) (A4B1, A2B2, A3B3, A5B5, A6B6, A7B3) or autoproc (73) (A7B7). Space groups were assigned using pointless and reflections were merged with aimless from the CCP4 suite (74, 75). All structures were solved by molecular replacement in Phaser (76) by searching for individual subunits with the following search models: Alphafold2-generated models of the chains (A3B3, A5B5), side-chain truncated models of the chains from pdb entry 8DA3 (A6B6), side-chain truncated models of the A6B6 chains (A4B1, A2B2, A7B7), and side-chain truncated models of the chains from pdb entry 8DAB (A7B3). Some structures were then rebuilt with phenix.autobuild (77) (A3B3, A6B6, A7B7, A7B3). Additional refinement was performed interactively in Coot (78) and in Phenix (79-82) (A2B2, A3B3, A5B5, A6B6, A7B7) or Buster (83, 84) with final refinement in Phenix (A4B1, A7B3). Final refinement included NCS restraints (A4B1) TLS parameters determined automatically in Phenix (A4B1, A3B3, A5B5, A6B6, A7B7, and A7B3). Model geometry was assessed with Molprobity (85) and contacting residues were identified with Pymol (Schrodinger, LLC) using a distance cutoff of 3.8 Å. Crystallographic software used in this project was compiled and maintained by SBGrid (86). Crystallographic data collection and refinement statistics along with PDB deposition codes are reported in table S1.

### Selection of representative cluster structures

To obtain structural representatives for each cluster, we initially attempted to crystallize all seven cluster structures along the diagonal of the specificity matrix in Figure 4A, along with two high-affinity cross-reactive complexes (A4B1 and A7B3). Among these, the crystal structures of A3B3, A5B5, A6B6, A4B1, and A7B3 were successfully solved. To expand our structural coverage, we selected additional cluster sequences that differed by a single amino acid substitution (one edit distance) from the original representative pairs. These variants were tested for binding and crystallization, leading to the successful structure determination of A2B2-L14^A^M for Cluster 2 and A7B7-L43^B^F for Cluster 7. For Cluster 1, an initial structure solution by molecular replacement at 1.73 Å resolution showed clear density for protein backbones, but most sidechain density was uninterpretable and the structure could not be refined. Closer inspection of the diffraction images identified satellite reflections consistent with incommensurate modulation of the crystal. In light of this result, we leveraged the A4B1 structure as a representative of cluster 1, as it differs from A1B1 by only a single amino acid substitution located outside the interface (fig. S20). Furthermore, we confirmed that the A4B1 structure aligns well with the backbone conformation of A1B1, validating its use as a pseudo-representative structure for Cluster 1.

### Surface plasmon resonance

Dissociation constants (K_D_) for Z-A/Z-B dimers were measured by surface plasmon resonance (SPR) on a BIAcore T100 instrument (GE Healthcare). Biotinylated Z-A chains were immobilized on a streptavidin-coated (SA) sensor chip (Cytiva), with a reference channel containing an unrelated protein. All experiments were performed in HBS-P+ buffer (Cytiva) supplemented with 0.5% BSA. Serial three-fold dilutions of analytes, starting from 50 μM, were injected at a flow rate of 50 μl/min, with an association phase of 120 s followed by a dissociation phase of 180-300 s. After each injection, the surface was regenerated with 0.1 M glycine (pH 2.5). Sensorgrams were analyzed using the BIAcore T100 evaluation software, and binding constants were determined by fitting to a steady-state affinity model.

### Selection probabilistic model (SPM)

We developed a statistical machine learning generative model to model all of the multi-round read count data simultaneously. The core quantity estimated by the model is the probability that any sequence is selected in a given round; the model is aware that output from one selection round is input to the next round. This core, estimated quantity can be thought of as the energy/fitness landscape for that round, and can be aggregated across rounds to obtain an overall energy/fitness function. We call our approach the selection probabilistic model (SPM). SPM is a generative model in that it models the data-generating process of multi-round selection experiments (depicted in Fig. S1A) by describing each round in two steps; (i) an update of the sequence distribution from the previous round, where the update arises from the selection process and (ii) generation/sampling of the observed sequence read counts from the updated sequence distribution in the first step, yielding a multinomial likelihood that can be maximized to estimate model parameters. For a detailed description refer to SI Section A. We define ${f_{r}}^{\theta }(x)$ as the inferred fitness for round r, with learned parameters θ, as the logarithm of the estimated selection probability (for each sequence and each round), and $f^{\theta }(x)$ as the global (over all rounds) fitness function. The energy is defined as negative fitness. Having inferred these fitness/energy landscapes, we use these for two down-stream applications: (1) epistasis analysis (SI Section C) and (2) simulation of coevolutionary trajectories based on the fitness landscape (SI Section D).

### SPM training and evaluation

Using the SPM, we learned a function ${f_{r}}^{\theta }(x)$ represented as a neural network, to predict the fitness associated with each selection round r for each sequence x (3,650,782 unique sequences in total). The optimal settings of the parameters θ were determined by maximizing the logarithm of the multinomial likelihood (described in SI Section B) over the sequences in each selection round. Our neural network, $f^{\theta }(x)$, comprised four fully connected hidden layers, each with a ReLU activation function (Fig. S1B). To stabilize the training, we applied regularization by weight decay (1e-5) with the Adam optimizer (87) and a dropout rate of 0.1. The hyperparameters, consisting of number of the hidden layers (4), hidden dimension (100), learning rate (0.001), batch size (10,000) and regularization strength, were chosen based on the model that produced the lowest MSE between the predicted counts and the experimental observation in a five-fold cross validation on the final selection round (while always using all previous rounds for training). We refer to SI Section B for the details of hyperparameter selection and model validation.

### Extraction of epistasis importance from SPM-predicted fitness landscape

We use two different types of epistatic characterizations for each epistatic term, (i) the epistatic *effect size*, and (ii) the epistatic *importance*. As is made more precise in Supplementary Information Section C, the effect size corresponds to the standard effect size one obtains in a linear additive model comprising all epistatic effects of all orders. For example, one epistatic effect size could be the parameter value in the linear additive model for position 2 being an A and position 5 being an L—a second order effect because it involves two positions. In contrast, the epistatic importance refers to how much changes in amino acids at specified positions can change the fitness. For example, one epistatic importance could be the maximum fitness minus the minimal fitness obtained by choosing the least and most favorable amino acids at position 2 and 5—hence importance is almost like defining the maximally achievable dynamic range at a set of positions, over all possible amino acids. Practically, we use effect size when we’re characterizing epistasis related to a particular variant (sequence), whereas when we seek to characterize epistasis for the entire fitness landscape, then importance is the relevant quantity because it is not anchored on any particular variants.

Briefly, we developed an efficient method to extract epistatic information, that is, the importance of different interaction terms; single site, pairwise, and all higher orders—from the learned sequence-to-fitness relationship. In this method, a linearized version of the learned fitness function is constructed that consists of the effect size of different terms (*e.g.*, single site, pairwise, or higher order). This method is related to the Walsh-Hadamard transform of the fitness function (88, 89). After computing the effect sizes of the linearized fitness function, we then define the epistasis importance of a term as the difference between maximum and minimum effect sizes of all possible configurations of amino acids for that term. This choice is inspired by the partial dependence-based variable importance measure for categorical values introduced in the work by Greenwell *et al.* (90). A detailed description of our epistasis-extraction method can be found in SI Section C.

### Simulating coevolutionary trajectories on the SPM-predicted fitness landscape

To extract the geometry of our estimated binding fitness landscape, we simulated coevolutionary trajectories through it. We simulated coevolutionary trajectories as paths from random points on the landscape that always increase the fitness. Specifically, each move in a trajectory considers all single-position edit distance mutations that increase the fitness, and selects one such single edit at random. This process iterates until no moves are left that increase the fitness, at which point we have arrived at a terminal state, namely, a local minimum. After simulating 10 million such trajectories, we compute the “accessibility” of each sequence as the fraction of trajectories that ended at that sequence. Accessibility of an energy well is defined as the accessibility of that well’s lowest-energy sequence. Such an analysis can provide insight into the ruggedness of landscape (the more local minima, the more rugged it is), as well as how different parts of sequence space differ (or not) in their accessibility. Although we simulated 10 million trajectories, fewer trajectories resulted in similar conclusions, suggesting that 10 million was sufficient. More details are provided in SI Section D.

### Energy landscape well depth, accessibility (width), and their statistical significance

In each energy landscape plot (such as the ones in Fig. 3F, fig. S8C and fig. S8D), each well has a specific variant that defines its energy minimum (shown with a colored dot). The width of a well is drawn proportional to the accessibility of that well, which can be seen by tracing out the energy barrier denoted by black lines to adjacent wells. For ease of visualization, we show only wells with the 20 most accessible sequences. For these 20, we chose a random ordering of the wells, with the exception of the natural interface which always centers the deepest energy well, using a random ordering for the rest. Later when we bootstrap this analysis to obtain statistical significance, each bootstrap data set has its own random ordering, thereby revealing that this ordering is unimportant to the major conclusions about differences in landscape geometry between synthetic and natural interfaces.

Although wells are shown adjacent to each other in these visualizations, namely with each well having one neighboring well to the left, and one to the right (other than the two wells on each end), in reality, each well has an energy barrier to every other well (and in particular to the other 19 wells in the plot). Consequently, we define (and show) the depth of an energy well as the minimal cumulative energy barrier that a coevolutionary trajectory needs to overcome from the representative sequence of this well to the representative sequence of any of the other 19 wells.

We use Dijkstra’s algorithm to find the path with the minimal energy barrier (details in SI Section D).

Having defined the width (accessibility) and depth, we can now define statistical tests related to these quantities in order to gauge if the differences between natural and synthetic interfaces are statistically significant. To do so, for each interface, we sampled 1,000 bootstrap NGS read data sets (each containing the original number of read counts in that data set). For each of these 1,000 data sets, we re-computed our energy landscapes, well accessibility and depths. Specifically, we trained an SPM to predict the fitness landscape––for each of these 1,000 data sets––with which to then simulate 10 million coevolutionary trajectories. We then performed a Mann-Whitney U test for the alternative hypothesis that the depth of the most accessible energy well in the natural interface is larger than 3 times the depth of the deepest energy well among the 20 most accessible wells in the synthetic interface (for 4 times larger, the results were not significant at level α=0.05). Similarly, we performed a Mann-Whitney U test for the alternative hypothesis that the accessibility of the most accessible energy well in the natural interface is larger than 5 times the accessibility of the most accessible energy well in the synthetic interface (for 6 times larger, the results were not significant at level α=0.05).

### Identification of seed sequences from coevolutionary trajectories

We require each seed sequence for a specific target sequence to have three properties: (i) weak but experimentally detectable binding affinity (with SPM-predicted fitness between 18.35 and 22.07 which are the average fitness of sequences with NGS read count of 5 and 20 respectively from the final selection round). (ii) high exclusivity to its strong-binding target sequence, where exclusivity is defined as the ratio of the number of trajectories passing through a given weak-binding sequence and arriving at a specific strong-binding target sequence to the total number of all trajectories passing through the weak-binding sequence (minimum exclusivity = 0.9). (iii) high contribution to its strong-binding target sequence, where contribution is defined as the ratio of the number of trajectories passing through a given weak-binding sequence and arriving at a specific strong-binding target sequence to the total number of all trajectories arriving at the target sequence (minimum contribution = 0.01). Intuitively, sequences with high exclusivity serve as checkpoints in the evolutionary paths to their corresponding target sequences, while sequences with high contribution act as the main origins of the evolutionary paths to their corresponding target sequences. We focused our analysis on seed sequences that are at least three single-point mutations away from their strong-binding sequences.

### Scoring chain pairing specificity and sequence-structure compatibility using Frame2seq

We compute Frame2seq (32) model negative pseudo log likelihoods (PLL) at the interface to assess predicted chain pairing specificity and sequence-structure compatibility. We refer to Frame2seq negative PLL values at the interface as interface scores throughout this work. Given experimentally solved structures and the amino acid sequence at framework positions as input, we introduce a mask at the interface positions and score the clustered library sequences at the masked indices to output interface scores as follows:

(Eq. S1) $$-\log\;p(x_{i}=x_{i}^{library}|x_{-i}^{library},S)$$

where i iterates over interface positions, $x^{library}$ is a clustered library sequence, $x_{-i}$ is the sequence x with a mask introduced at i, S is structure. We predict chain pairing specificity by scoring all Z-A and Z-B chain combinations on corresponding structures. We predict sequence-structure compatibility by scoring all clustered library sequences on all six experimentally solved structures.

### Extraction of structure-conditioned epistasis importance using Frame2seq

To extract structure-specific epistasis importance, we utilize the aforementioned epistasis importance extraction method (details described in SI Section C) but substitute the fitness function $f^{\theta }(x)$ from SPM with an approximate structure-conditioned fitness function $f_{F2s}^{\theta }(x\;|\;S)$ constructed using Frame2seq (where S represents input structural information). We use Frame2seq with published pre-trained weights (32) without fine-tuning. Details of how this function is constructed are provided in SI Section E, and our approximations are validated empirically in SI Section F.

## Supplementary Material

Supplementary tables S1 S2

adx6931_Supplementary Materials

adx6931_Reproducibility Checklist_seq1_v3

Supplementary Text

Table S1 to S2

Figures S1 to S21

References (94 – 101)

MDAR Reproducibility Checklist
