# Supplementary material for: Structural ontogeny of protein-protein interactions
Source: Science. Author manuscript; Available in PMC 2026 Feb 13. (PMC12904254; doi:10.1126/science.adx6931)
Supplement: adx6931_Supplementary Materials [file NIHMS2128659-supplement-adx6931_Supplementary_Materials.pdf]

Supplementary Materials for

Structural ontogeny of protein-protein interactions

Authors:
Aerin Yang[1]†, Hanlun Jiang[2]†, Kevin M. Jude[1,4]†, Deniz Akpinaroglu[5,6], Stephan Allenspach[2]†, Alex Jie Li[5,6], James Bowden[2], Carla Patricia Perez[7], Liu Liu[1], Po-Ssu Huang[8], Tanja Kortemme[5,6,9], Jennifer Listgarten[2,3,5]*, K. Christopher Garcia[1,4]*

*** Corresponding authors:** jennl@berkeley.edu, kcgarcia@stanford.edu

This PDF file includes:

Supplementary Information
Figs. S1 to S21
Tables S1 to S2
References (94 – 101)

Supplementary Information

A. Selection probabilistic model (SPM)

For our analysis, we developed a new method that directly describes the data-generating process of multi-round selection experiments. This process (shown in Fig. S1 A) corresponds to a cascade of multiple rounds that each consist of two steps: (i) a selection step where the current sequence distribution is updated, and (ii) an observation step where sequences are drawn from the updated sequence distribution.

We start with the observation step in round r. A sequence x is randomly chosen from the round-r sequence distribution $p_r(x)$ resulting in a read count of $N_r(x)$ observed for this sequence and in a total number of sequencing read counts, $N_r^{tot} = \sum_{x \in X} N_r(x)$, observed over the set of all sequences X. This process is described by a multinomial likelihood

$$p(D_r|\theta) = \text{Multinomial}([N_r(x)]_{x\in X} \mid [p_r(x)]_{x\in X}, N_r^{tot})$$

where $D_r \equiv \{x, N_r(x)\}_{x\in X}$ corresponds to the data (*i.e.*, set of counts observed for the sequences) of round r and $[\dots]_{x\in X}$ is used to denote a set of counts or probabilities ordered by their corresponding sequences. The round-r sequence distribution $p_r^\theta(x)$ is unknown and must be modeled − indicated by the to-be-determined model parameters θ − as we will describe in detail in the next paragraph. Because the collections of counts in different rounds are independent, the likelihood over all rounds factorizes to

$$p(D|\theta) = \prod_{r=0}^{R} p(D_r|\theta) = \prod_{r=0}^{R} \text{Multinomial}([N_r(x)]_{x\in X} \mid [p_r(x)]_{x\in X}, N_r^{tot}) \qquad \text{(A1)}$$

where $D \equiv \{D_r\}_{r\in\{0,\dots,R\}}$ is the data (*i.e.*, the set of counts) over all rounds and R is the number of selection rounds. Note that Eq. (A1) includes the initial round ($r = 0$) prior to the first selection round ($r = 1$) assuming that counts have already been obtained for the sequence distribution prior to any selection, which was the case in our experiments.

After discussing the observation step at the end of a round, we turn now to the selection step at the beginning of a round. This selection step can be described as a selection-induced update of a sequence distribution $p(x)$ (prior to selection) to a post-selection sequence distribution $p(x|\text{selected} = \text{True})$. Mathematically, we can express this update using Bayes' theorem

$$p(x|\text{selected} = \text{True}) = \frac{p(\text{selected} = \text{True}|x)p(x)}{\sum_{x' \in X} p(\text{selected} = \text{True}|x')p(x')} \tag{A2}$$

where $p(\text{selected} = \text{True}|x)$ corresponds to the probability that a sequence is selected, and we will call these the selection probabilities. For each selection round r, we identify $p(x|\text{selected} = \text{True}) \rightarrow p_r^\theta(x)$ and $p(x) \rightarrow p_{r-1}^\theta(x)$, and rewrite Eq. (A2) as

$$p_r^\theta(x) = \frac{\eta_r^\theta(x)p_{r-1}^\theta(x)}{\sum_{x' \in X} \eta_r^\theta(x')p_{r-1}^\theta(x')} \tag{A3}$$

defining the selection probabilities for a sequence x to be selected in round r as $\eta_r^\theta(x)$. Starting from an initial distribution prior to any selection (*i.e.*, prior to round $r = 1$), $p_0^\theta(x)$, we can iteratively obtain the sequence distributions $p_r^\theta(x)$ in all selection rounds using the selection probabilities $\eta_r^\theta(x)$. Thus, the task of determining $p_r^\theta(x)$ turns into the task of determining $\eta_r^\theta(x)$. Eq. (A3) illustrates that the probability of drawing a sequence x in round r, $p_r^\theta(x)$, depends not only $p_{r-1}^\theta(x)$ but also on $p_{r-1}^\theta(x')$ for all other sequences $x' \in X$. In contrast, the probability of a sequence x to be selected in round r, $\eta_r^\theta(x)$, does only depend on the sequence x itself and not on any other sequence. If the initial distribution $p_0^\theta(x)$ is unknown, we have to either approximate it (*e.g.*, as a uniform distribution) or determine it in addition to $\eta_r^\theta(x)$.

As $p_r^\theta(x)$ in Eq. (A3) is invariant under rescaling of $\eta_r^\theta(x)$ with any non-zero scalar value, we chose to alternatively express the selection probabilities $\eta_r^\theta(x)$ as

$$\eta_r^\theta(x) = \exp\left(f_r^\theta(x)\right) \equiv \exp\left(-E_r^\theta(x)\right)$$

where $f_r^\theta(x)$ is learned fitness of sequence ~~x~~x for round r. We define the energy, $E_r^\theta(x)$, of a sequence x in round r as its negative fitness (*i.e.*, $E_r^\theta(x) \equiv -f_r^\theta(x)$) (*47*). Note that the invariance of Eq (A3) under rescaling $\eta_r^\theta(x)$ turns into an invariance under shifting $f_r^\theta(x)$. Consequently, the (absolute) energy is known up to an additive constant. For this reason, although we cannot directly compare energies, $E_r^\theta(x)$, between two landscapes such as natural and synthetic, we can compare relative energies which are invariant to this additive constant. Said another way: during the selection of yeast-display libraries, the differences in binding affinities between the sequences, rather than their absolute binding affinities, directly affect the observed read counts in the NGS data. Since SPM explicitly models the selection experiments, it is invariant under any global shifts of the absolute binding energies. Mathematically, this means that adding an arbitrary constant to

the absolute energies (i.e., shifting them by an arbitrary value) of all the sequences results in the same likelihood of the NGS data. Consequently, the determined absolute energies cannot be compared across NGS datasets of different libraries. However, note that differences between the absolute energies are preserved under constant shifts. As the logarithm of the absolute energy of a sequence is proportional to its selection probability, differences in the absolute energies relate to ratios between selection probabilities. As one can compare ratios of selection probabilities of different sequences across NGS datasets, one can also compare energy differences (i.e., 'relative energy') across datasets.

In this article, we parameterize $f_r^\theta(x)$ as the r-th output of a neural network $f^\theta(x): \mathbb{R}^{A\times L} \to \mathbb{R}^R$ that maps a one-hot encoded sequence x — with number of library positions $L = 11$ and with amino acid alphabet size $A = 5$ — to its fitness values in all rounds. Moreover, we assume that the initial distribution $p_0^\theta(x)$ is uniform. The parameters of the neural network $f^\theta(x)$ can be learned by minimizing the negative logarithm of the likelihood in Eq. (A1), $L_D(\theta) \equiv -\log\big(p(D|\theta)\big)$, for all sequences for which any counts have been observed in the NGS data.

Our method presented here can be considered a generalized probabilistic description of multi-round selection experiments that includes for example the method presented in (*94*) as a special case where a domain-specific selection probability has been employed to study protein stability. Moreover, our method shares some aspects with the method presented in (*95*) designed for the analysis of data collected in directed evolution experiments where the set of sequence can change from one round to the next due to mutations.

B. SPM training and validation

The collection of the sequencing data from pre-selection libraries as well as that from each round of selection contains 3,650,782 unique sequences. For each of these sequences, we assign a count of 0 for the selection rounds where the sequence is not observed. This processing produces a dataset:

$$D \equiv \big\{x_i, \{N_r(x_i)\}_{r\in\{0,1\ldots 5\}}\big\}_{i\in\{1,\ 2,\ \ldots\ ,\ 3,650,782\}}$$

where i is the index of each unique sequence, x_i is a unique concatenated, one-hot encoded sequence of Protein A and Protein B, r is the index of selection round except for $r = 0$ denoting the pre-selection round, and $N_r(x_i)$ is the read count of sequence x_i from selection round r. We

divided the dataset evenly into five folds by a stratified splitting based on the last selection round where a given sequence has a positive count.

We applied SPM for modeling the sequencing data from the muti-round selection experiment, and determined the parameters θ of the neural network $f^{\theta}(x)$ with maximum likelihood estimation. To stabilize the training, we applied regularization by using weight decay (1e-5) in the Adam optimizer and a dropout rate of 0.1. We scanned the following hyperparameters (with the tested values denoted in the parentheses): number of the hidden layers (1, 2, 3 and 4), hidden dimension (50, 100, 200 and 300), learning rate (0.01, 0.001 and 0.0001) and batch size (10,000, 100,000). The models trained with different hyperparameters were evaluated and ranked by the MSE between predicted counts and observed counts in the final selection round for the sequences in the held-out test fold from a five-fold cross-validation. We chose the optimal set of hyperparameters as: number of the hidden layers (4), hidden dimension (100), learning rate (0.001), batch size (10,000).

C. Characterizing epistasis in the SPM-predicted fitness landscape

We present a general method to extract epistatic characteristics of a functions defined on discrete states (*e.g.*, function of sequences such as for the protein fitness landscape used herein). Our method obtains an expression for a linearized version of any arbitrary such function, and this linearization can be exact if desired (by using epistatic terms up to the maximal order, namely the length of the protein). This method is intimately related to the Walsh-Hadamard transform (*88*, *89*) and has been developed independently from an analogous method presented in (*96*).

In the main manuscript, we use two different types of epistatic characterizations for each epistatic term, (i) the epistatic *effect size*, and (ii) the epistatic *importance*. As will be made more precise below, the effect size is the standard effect size one obtains in a linear additive model (here for some fixed set of positions and amino acids at those positions), while the importance refers to how much a given epistatic term can change the fitness (here for some fixed set of positions, over all possible amino acids), which is related to the optimal amino acid, and least optimal amino acid at the relevant positions—this can be thought of like a dynamic range resulting from all epistatic effects at those positions. Note that we use effect size when we're characterizing epistasis related to a particular variant (sequence). However, when we want to characterize epistasis for the entire fitness landscape, then importance is the relevant quantity.

Let $X = X_1 \times X_2 \times \ldots \times X_L$ be the space of all states that consists of L one-dimensional categorical subspaces X_l with cardinality $C_l = |X_l|$ for each location $l \in \Lambda \equiv \{1, 2, \ldots, L\}$. In our case, C_l will always be equal to the number of amino acids allowed at each position, namely for our synthetic libraries, 5. Consequently, going forward, we use $C_l = C$. States $x \in X$ consist of features $\{x_1, x_2, \ldots, x_L\}$ $-$ $i.e., x = (x_1, x_2, \ldots, x_L)$ $-$ where a feature x_l on location l can take any of the ~~C~~ C categorical values, and thus $|X| = \prod_{l \in L} C_l = \prod_{l \in L} C = C^L$ different states can be realized in the entire state space X. In this article for example, x corresponds to a sequence, the locations to the manipulated residues, and the features of one location to the amino acids that can be placed on a residue. Thus, there are $L = 11$ locations (*i.e.*, residues here) that each can be populated by any of the 5 amino acids $\{F, I, L, M, V\} = X_l$ so that $C = C_l = 5$ for any location l and thus $|X| = 5^{11}$.

We define a term $t \equiv \{l_1, l_2, \ldots, l_K\}$ of order K as a set of K unique locations $l_1, \ldots, l_K \in \Lambda$. For example, $\{4\}$ is a term of order $K = 1$, $\{3,5\}$ is a term of order $K = 2$, and $\{\}$ is the zero-order term ($K = 0$). As there are $\binom{L}{K} \equiv L!/[(L-K)!\,K!]$ ways to assemble a set of K unique locations out of a total number of L locations, there are $\binom{L}{K}$ terms of order K and the set of all terms is given by the powerset $P[\Lambda] = P[\{1,2, \ldots, L\}] = \{\{\}, \{1\}, \{2\}, \ldots, \{L\}, \{1,2\}, \ldots, \{1, L\}, \ldots\}$ containing $\sum_{K=0}^{L} \binom{L}{K} = 2^L$ terms. For example for $L = 2$, there are $2^2 = 4$ terms; $\{\}$ (with $K = 0$), $\{1\}$ (with $K = 1$), $\{2\}$ (with $K = 1$), and $\{1, 2\}$ (with $K = 2$).

For each term $t \equiv \{l_1, l_2, \ldots, l_K\}$ one can construct *term-states* that we denote as $x[t] = (x_{l_1}, x_{l_2}, \ldots, x_{l_K}) \in X_{l_1} \times X_{l_2} \times \ldots \times X_{l_K} \equiv X_t$ and there are $|X_t| = \prod_{l \in t} C_l$ many term-states for one term. For any location l, there are C^K term-states for a term of order K and thus $\sum_{K=0}^{L} \binom{L}{K} C^K = (C+1)^L$ term-states in total. For example if $L = 2$ with $x = (x_1, x_2)$ and $C = 2$ so that $x_i \in \{A, B\}$, there are $3^2 = 9$ term-states; 1 for $K = 0$, 4 for $K = 1$, and 4 for $K = 2$. $x[\{\}] = ()$ has (for any C) only a single term-state $()$. $x[\{1\}] = (x_1)$ has two term-states (A) and (B). $x[\{2\}] = (x_2)$ has also two term-states (A) and (B). $x[\{1,2\}] = (x_1, x_2)$ has four term-states (A, A), (A, B), (B, A), and (B, B).

Consider the function $f: X \longrightarrow \mathbb{R}$ that maps a state (*e.g.*, a sequence) $x \in X$ to a scalar value (*e.g.*, the fitness of the sequence). We can decompose this function into a linear additive model, that is, into contributions of all different terms in x as

$$f(x) = \phi_0 + \sum_{l} \phi_l(x_l) + \sum_{l_1<l_2} \phi_{l_1,l_2}(x_{l_1}, x_{l_2}) + \ ... \ = \sum_{t\in P[\Lambda]} \phi_t(x[t])$$

where $\phi_t\colon X_t \longrightarrow \mathbb{R}$ is the *contributor* of term t. A contributor of a term maps each of the term's states to their corresponding coefficients, which we refer to as the *effect* or *effect size*, since it corresponds to a standard fixed effect in a linear additive model. We note that one can express contributors of, for example, first order terms alternatively as

$$\phi_l(x_l) = \sum_{i=1}^{C_l} a_l^i \rho_{OHE}(x_l)^i$$

where a_l^i is the coefficient/effect associated with i-th term-state of term x_l and $\rho_{OHE}(x_l)^i$ is the i-th component of the one-hot encoding (OHE) of x_l. Thus, if the term-states of x_l are $\{x_l^1, \dots, x_l^{C_l}\}$, we find that $\phi_l(x_l = x_l^i) = a_l^i$. Note that ϕ_0 is the effect size of the empty term {}, sometimes called a bias or offset term. The contributor of a K-th order term, $\phi_t(x[t])$, in general has C^K different effects/coefficients, one for each term-state of $x[t]$. As there are $(C+1)^L$ term-states, there are a total of $(C+1)^L$ effects/coefficients in this case.

Our goal is to determine effects (*i.e.*, effect sizes) from the function values $f(x)$, which are assumed to be known and noiseless (*e.g.,* as defined by a trained neural network). One way to achieve this goal is through marginalization as we will show next. We define the marginal of the feature x_l on location l as the average of the function $f(x)$ over all features on all locations except on location l itself

$$M_l(x_l) = \frac{1}{|X_1|} \sum_{x_1\in X_1} \cdots \frac{1}{|X_{l-1}|} \sum_{x_{l-1}\in X_{l-1}} \frac{1}{|X_{l+1}|} \sum_{x_{l+1}\in X_{l+1}} \cdots \frac{1}{|X_L|} \sum_{x_L\in X_L} f(x) = \frac{1}{|X_{\Lambda\backslash\{l\}}|} \sum_{\{x_j\}_{j\in\Lambda\backslash\{l\}}} f(x)$$

where $\Lambda\backslash\{l\}$ denotes all the locations except l, and $\{x_j\}_{j\in\Lambda\backslash\{l\}}$ denotes the set of all feature-values of the features on locations $\Lambda\backslash\{l\}$. Analogously, one can define the marginal over a term t as

$$M_t(x[t]) = \frac{1}{|X_{\Lambda\backslash t}|} \sum_{\{x_l\}_{l\in\Lambda\backslash t}} f(x) \qquad \text{(C1)}$$

where $\Lambda\backslash t$ denotes all the locations that do not appear in term t, and $\{x_l\}_{l\in\Lambda\backslash t}$ denotes the set of all feature-values of the features on locations $\Lambda\backslash t$. So in simple words; the marginal over a term in Eq. (C1) corresponds to an average over all features on locations not appearing in term t.

As the number of all effects is larger than the number of states, there are extra degrees of freedom in the effects. For example for any location l, there are $(C+1)^L$ term-states and thus $(C+1)^L$ effect as previously mentioned, while there are C^L states and thus C^L function values $f(x)$. To fix these extra $[(C+1)^L - C^L]$ degrees of freedom, one can choose a gauge (a mathematical concept used to reduce the redundant degrees of freedom of a system). A convenient gauge that will simplify the determination of contributors from marginals, is the zero-sum gauge (*97*) that uses the following constraint to reduce the degrees of freedom,

$$0 = \sum_{x_l \in X_l} \phi_{t\cup\{l\}}(x[t \cup \{l\}]) = \sum_{x_l \in X_l} \phi_{t,l}(x[t], x_l)$$

for any location any $l \in \Lambda$ and any term $t \in P[\Lambda \backslash \{l\}]$. Use of this gauge (constraint) removes the redundant degrees of freedom by requiring that the sum over the feature-values of any of its features is equal to zero. After fixing the gauge to be zero-sum, we end up with C^L independent effect sizes describing the C^L degrees of freedom that the function values $f(x)$ can take.

One can show that the marginal of a term t can be written as

$$M_t(x[t]) = \sum_{p \in P[t]} \phi_p(x[p]) = \phi_t(x[t]) + \sum_{p \in (P[t] \backslash \{t\})} \phi_p(x[p]) \qquad \text{(C2)}$$

with contributors ϕ expressed in the zero-sum gauge, where $(P[t] \backslash \{t\})$ is the powerset of term t not including the term itself. Note that all terms in $(P[t] \backslash \{t\})$ have a lower order than the order of term t. This means that after determining the marginals from the function values $f(x)$, we can determine the contributors ϕ (expressed in the zero-sum gauge) iteratively in increasing order of terms from the marginals by rearranging Eq. (C2) to

$$\phi_t(x[t]) = M_t(x[t]) - \sum_{p \in (P[t] \backslash \{t\})} \phi_p(x[p]).$$

Although effect sizes are useful for informing on the effect of specific amino acids at specific positions, we may wish to characterize the importance of epistasis more generally at some set of positions, that is, over all possible amino acids. To do so, we define *importance* as the maximum range of the effect sizes achievable for a term t as

$$I(t) = max(\{\phi_t(x[t])\}) - min(\{\phi_t(x[t])\}).$$

This definition is inspired by the partial dependence-based variable importance measure for categorical values introduced in the work by Greenwell *et al.* (*90*). Another gauge-invariant term-importance would for example be the variance of the effect sizes of a term; $var(\{\phi_t(x[t])\})$.

In summary, our algorithmic recipe is to determine (1) the marginals, then (2) the effect sizes in the zero-sum gauge from the marginals, and (3) the importance of each term from the effect sizes of all terms. Both effect sizes and term-importance values characterize different aspects of epistasis, we refer to them as "epistasis effect sizes" and "epistasis importance", respectively, in the manuscript. While an epistasis effect size is sequence-specific description of local epistatic information, epistasis importance is a sequence-agnostic characterization of the global epistatic landscape.

We applied this method to the learned fitness function f^{θ} (discussed in SI Section A) to extract the epistasis importance for all terms. Note that a sequence input to f^{θ} consists of residues on protein A and protein B with L_A and L_B locations each. Consequently, intra-protein terms, which only include locations on a single protein, only exist up to an order of $min(L_A, L_B)$. In Fig. 6 we only display epistasis importance up to the maximal order for intra-protein terms.

D. Simulating coevolutionary trajectories to characterize the SPM-predicted fitness landscape geometries

We wanted to ask several questions about the SPM-estimated energy landscape that underlies the protein interface. In particular, what is the shape of this landscape? Does it have many local minima that during natural or directed evolution (DE), we are likely to get stuck in? Is there a single binding mode that is strongly favored, perhaps evidenced by a wide (local) minimum? How accessible are various desirable sequences, such as the best binding sequences, from arbitrary starting points (as we may face when running DE)? Was it important to assay the entire combinatorial library, or would DE have yielded strong binders much more efficiently? Such questions are effectively characterizations of the energy landscape's shape; while there are derivative-based tools to do so easily in the continuous setting, the protein sequence space is inherently discrete and so we rely instead on (many) trajectories run over the landscape to get a sense for its curvature. This idea is inspired by (*98*, *99*); we typically do not have access to enough data to analyze the energy landscape globally and so usually can only reason based on the specific DE paths observed, but here, we may simulate the process underlying DE and so make statements about what might happen on average (with initialization point and measurement stochasticity averaged out).

To simulate coevolutionary trajectories for a binding interface, we require an energy landscape E: $X \rightarrow \mathbb{R}$. Importantly, we must be able to query the energy landscape at *any* sequence in a pre-

defined space X; in other words, the landscape must be dense. This suggests that learned surrogate landscapes are likely to be the main viable option. We must also believe that the shape of the landscape is reasonable as this is what will determine where the trajectories flow. In particular, querying a learned energy landscape at points beyond its training distribution may yield unpredictable and inconsistent behavior. This makes our learned energy landscape particularly well-suited for this task, as it is trained on sequencing read counts gathered from experiments that included all library sequences at the chosen binding interface, such that there isn't any missing data in a distributional coverage sense. Other evolution simulation approaches such as Teufel *et al.* 2017 (*100*) focus on reducing the number of costly queries to a fitness landscape needed to draw conclusions; we do not face this problem due to the unique nature of our combinatorically-complete assay and read-count dataset.

Given an energy landscape E (*i.e.*, the negative fitness landscape learned from the observed counts), we run a single downhill trajectory by picking a starting sequence x_0 with uniform probability from X. We then collect all the neighbors of x_0 defined as sequences with edit distance one (single mutants), N_0. From N_0 we consider only sequences with a favorable mutation (energy decreases relative to x_0) and pick a sequence x_1 with uniform probability from $\{x \in N_0 : E(x) < E(x_0)\}$. More generally, we select sequence $x_{i+1} \sim Unif(\{x \in N_i : E(x) < E(x_i)\})$; $N_i = \{x \in X : H(x, x_i) = 1\}$, where $H(a, b)$ is the Hamming distance between sequence a and sequence b. A trajectory is defined as a series of sequences with decreasing energy $\{x_0, x_1, \ldots, x_t : E(x_0) > E(x_1) > \cdots > E(x_t)\}$ and terminates at x_t when there are no favorable mutations to be made, *i.e.*, when we arrive at a local minimum. We run, in parallel, 10 million trajectories each starting from a random sequence, for each interface.

Note that this algorithm is greedy in the sense that we always take a step that improves the energy, but that we do not discriminate between a sequence that improves the energy and the sequence that improves the energy the *most*. In other words, we do not consider (biologically plausible) trajectories that tolerate temporary decreases in fitness, and also do not assume that the optimal mutation is found at each mutation step either. We chose to only consider single position mutations at each evolution step for computational tractability; in nature, n-position mutations can and do occur though often at lower frequencies. However, modeling arbitrary numbers of mutations in one step requires considering combinatorically increasing numbers of neighbors. In our current

setup, we only have to consider $O(A * L)$ neighbors at each step, with A denoting the size of amino acid alphabet and L denoting the number of library positions.

To characterize the simulated coevolutionary trajectories, we perform two main analyses. First, we determine which sequences are local minima in the energy landscape and analyze their accessibility by summing up the number of trajectories that end in each sequence. This provides insight into how many minima the energy landscape has and the relative accessibilities of each one; namely, which minima are favored by the curvature of the landscape? Second, we add up visit counts across all sequences, now including those passed through along trajectories as well, and obtain an accessibility metric that is more general. With this, we can understand which sequences are highly accessible and so important as waypoints, though not the strongest binders, as evolution progresses. Together, these provide a way of understanding the shape of the energy landscape via the kinds and amounts of flows through it.

Separately, we characterize the well depths in the energy landscape by computing the energy barriers between each pair of wells. The energy barrier between a source sequence and a target sequence is computed as the minimum uphill distance traveled to get from the source to the target using only single mutations (non-negative). Assuming that evolution takes the most energetically favorable single-mutation path between two sequences, we'd like to characterize just how difficult and hence how "likely" evolution from the source to the target would be on our energy landscape. We formulate this problem algorithmically as shortest path finding, where the metric is the sum of energy increases along each mutation in the path, and implement Dijkstra's algorithm (*101*). Formally, we run Dijkstra's algorithm on the Hamming-distance 1 graph, where each node is a valid sequence and is connected to all other sequences a single mutation away, and the directed edge between two sequences is max(0, E(target) - E(source)), where E(target) and E(source) are the energies of target and source sequences respectively. It should be noted that energy barriers are not generally symmetric; a path between two sequences may incur a substantial energy barrier in one direction and a much smaller, or even zero, energy barrier in the reverse direction.

E. Extracting structure-conditioned pairwise epistasis importance using Frame2seq

Let S encapsulate both the backbone structure as well as the framework sequence of an input structure. We define our structure-conditioned fitness function as

$$f(x) = \log p(x \mid S)$$

For notational simplicity, we will also write $p_S(x) = p(x \mid S)$. To compute epistasis from this fitness function, we need to compute the *marginals* of this function as defined in SI Section C, or

$$\begin{aligned} M_t(x[t]) &= \frac{1}{|X_{\Lambda\setminus t}|}\sum_{x\in X_{\Lambda\setminus t}} f(x) \\ &= \frac{1}{|X_{\Lambda\setminus t}|}\sum_{x\in X_{\Lambda\setminus t}} \log p_S(x) \qquad \text{(D1)} \end{aligned}$$

where $\Lambda = \{1, \dots, L\}$ is the set of available residue positions. Naively, we could compute these marginals by scoring all possible sequences in X, but directly scoring this many sequences with Frame2seq is computationally intractable. We instead utilize a set of useful approximations to significantly reduce the number of Frame2seq evaluations while retaining informative epistasis information.

For any particular term t, we can utilize conditional probabilities to rearrange (D1) as follows:

$$\begin{aligned} M_t(x[t]) &= \frac{1}{|X_{\Lambda\setminus t}|}\sum_{x\in X_{\Lambda\setminus t}} \log p_S(x) \\ &= \frac{1}{|X_{\Lambda\setminus t}|}\sum_{x\in X_{\Lambda\setminus t}} \left(\log p_S(x[\Lambda\setminus t] \mid x[t]) + \log p_S(x[t])\right) \\ &= \log p_S(x[t]) + \frac{1}{|X_{\Lambda\setminus t}|}\sum_{x\in X_{\Lambda\setminus t}} \log p_S(x[\Lambda\setminus t] \mid x[t]) \qquad (D2) \end{aligned}$$

Frame2seq gives us access to us a set of probability distributions $\hat{p}_i(x \in \mathcal{A} \mid S, x[t])$, the per-position probabilities over the full amino acid alphabet $\mathcal{A}$ conditioned on S and $x[t]$. Such functions are computed by feeding Frame2seq [MASK] tokens at sequence positions not being conditioned on (e.g. those specified by S and $x[t]$) and taking the softmax of the outputted logits at position i.

We first show how to approximate the term outside the summation in (D2) using autoregressive decompositions. We can specify a particular autoregressive decomposition via a permutation σ which maps sequence positions to a specific autoregressive order. For this particular order,

$$\begin{aligned} p_\sigma(x[t] \mid S) &\approx \prod_{i\in t} \hat{p}_{\sigma(i)}\left(x_{\sigma(i)} \mid S, \{x_{\sigma(j)} \mid j < i\}\right) \\ \log p_\sigma(x[t] \mid S) &\approx \sum_{i\in t} \log \hat{p}_{\sigma(i)}\left(x_{\sigma(i)} \mid S, \{x_{\sigma(j)} \mid j < i\}\right) \qquad (D3) \end{aligned}$$

where the approximation arises because we approximate the effect of marginalizing over unscored positions $\{x_k,\ k \in \Lambda \backslash t\}$ by inputting mask tokens at those positions.

Computationally, we compute the log quantity (D3) as follows: for a given decoding order σ and decoding step i, we feed Frame2seq

- S (backbone structure and framework sequence)
- $\{x_{\sigma(j)} \mid j < i\}$ (the sequence of the previously scored residues)
- [MASK] at $X_{\sigma(k_1)},\ k_1 \geq j$ (unscored residues in t)
- [MASK] at $X_{k_2},\ k_2 \in \Lambda \backslash t$ (interface residues not in t)

and compute the log probability of identity $x_{\sigma(i)}$ at position $\sigma(i)$ from the softmax of the logits at position $\sigma(i)$. The sum over all decoding steps $1 \leq i \leq L$ then forms the estimate for the quantity $\log p_\sigma(x[t] \mid S)$.

Since there is no universal or special autoregressive decomposition, we approximate $\log p_S(x[t])$ as the average over all autoregressive decompositions possible for term t as done in (D3):

$$\begin{aligned}\log p_S(x[t]) &= \log p(x[t] \mid S) = \frac{1}{|\mathrm{Sym}(t)|} \sum_{\sigma \in \mathrm{Sym(t)}} \log p_\sigma(x[t] \mid S) \\ &\approx \frac{1}{|\mathrm{Sym}(t)|} \sum_{\sigma \in \mathrm{Sym}(t)} \sum_{i \in t} \log \hat{p}_{\sigma(i)}\left(x_{\sigma(i)} \mid S, \{x_{\sigma(j)} \mid j < i\}\right)\end{aligned}$$

where $\mathrm{Sym}(t)$ is the set of all permutations that can be constructed from the positions appearing in term t and thus corresponds to the set of all autoregressive decompositions possible for term t. When the order of t is small (e.g. $|t| \leq 2$ in this work), there are few such decompositions and computation is tractable via logit caching.

We next show how to approximate the term within the summation in (D2). As full autoregressive decomposition over all paths is computationally intractable in this case, we opt to compute a pseudo-likelihood-inspired quantity instead:

$$\log p_S(x[\Lambda \backslash t] \mid x[t]) \approx \sum_{j \in \Lambda \backslash t} \log \hat{p}_j\left(x_j \mid S, x[t]\right)$$

Notably, the per-position nature of this approximation allows us to reduce the summation in (D2) to

$$\begin{aligned}
&\frac{1}{|X_{\Lambda\backslash t}|}\sum_{x\in X_{\Lambda\backslash t}} \log p_S(x[\Lambda\backslash t] \mid x[t]) \\
&= \frac{1}{|X_{\Lambda\backslash t}|}\sum_{x\in X_{\Lambda\backslash t}}\sum_{j\in\Lambda\backslash t} \log \hat{p}_j\big(x_j \mid S, x[t]\big) \\
&= \frac{1}{|X_{\Lambda\backslash t}|}\sum_{j\in\Lambda\backslash t}\sum_{x_j\in\mathcal{A}_l} |X_{\Lambda\backslash(t\backslash\{j\})}| \log \hat{p}_j\big(x_j \mid S, x[t]\big) \\
&= \frac{1}{|\mathcal{A}_l|}\sum_{j\in\Lambda\backslash t}\sum_{x_j\in\mathcal{A}_l} \log \hat{p}_j\big(x_j \mid S, x[t]\big)
\end{aligned}$$

In words, we average the log-probabilities over the library amino acid alphabet per-position, then sum together each positional term to yield our desired quantity. In summary, the computation of the marginal for $x[t]$ becomes

$$\begin{aligned}
M_t(x[t]) \quad &\approx \frac{1}{|\mathrm{Sym}(t)|}\sum_{\sigma\in\mathrm{Sym(t)}}\sum_{i\in t} \log \hat{p}_{\sigma(i)}\big(x_{\sigma(i)} \mid S, \{x_{\sigma(j)} \mid j < i\}\big) \\
&+ \frac{1}{|\mathcal{A}_l|}\sum_{j\in\Lambda\backslash t}\sum_{x_j\in\mathcal{A}_l} \log \hat{p}_j\big(x_j \mid S, x[t]\big)
\end{aligned}$$

Once we compute the marginals as above, we follow the same methodology as in SI Section C (starting at Equation C2) to compute effect sizes and ultimately epistasis importance.

F. Empirical validation of Frame2seq approximations

As a sanity check, we approximate the base fitness function as the summation over computed effect sizes of order $|T| \leq 2$

$$\hat{f}(x) = \phi_0 + \sum_{l} \phi_l\,(x[\{l\}]) + \sum_{l_1<l_2} \phi_{\{l_1,l_2\}}\,(x[\{l_1,l_2\}])$$

and compare it to the Frame2seq interface score

$$\log \hat{p}_S(x) = \sum_{i} \log \hat{p}_i(x_i \mid S)$$

which is the sum of Frame2seq's per-position log-likelihood estimates over the interface residues when the entire interface is masked. While we could choose to compare against the full autoregressive likelihood, we know that the Frame2seq interface score correlates well with experimental data, and hence we choose to compare against it instead.

We compare the two fitness functions when evaluated on library sequences conditioned on each cluster crystal structures (fig. S21) and find they give strong correlations. We also note that they are not perfectly correlated, which is also a good sanity check as we cannot extract epistasis from the Frame2seq interface score under the SPM framework due to its per-position nature.

Visualization

PyMOL

GraphPad Prism 10

BioRender (https://biorender.com/)

WebLogo 3 (http://weblogo.threeplusone.com/)

Igraph

PyCirclize

Matplotlib

seaborn

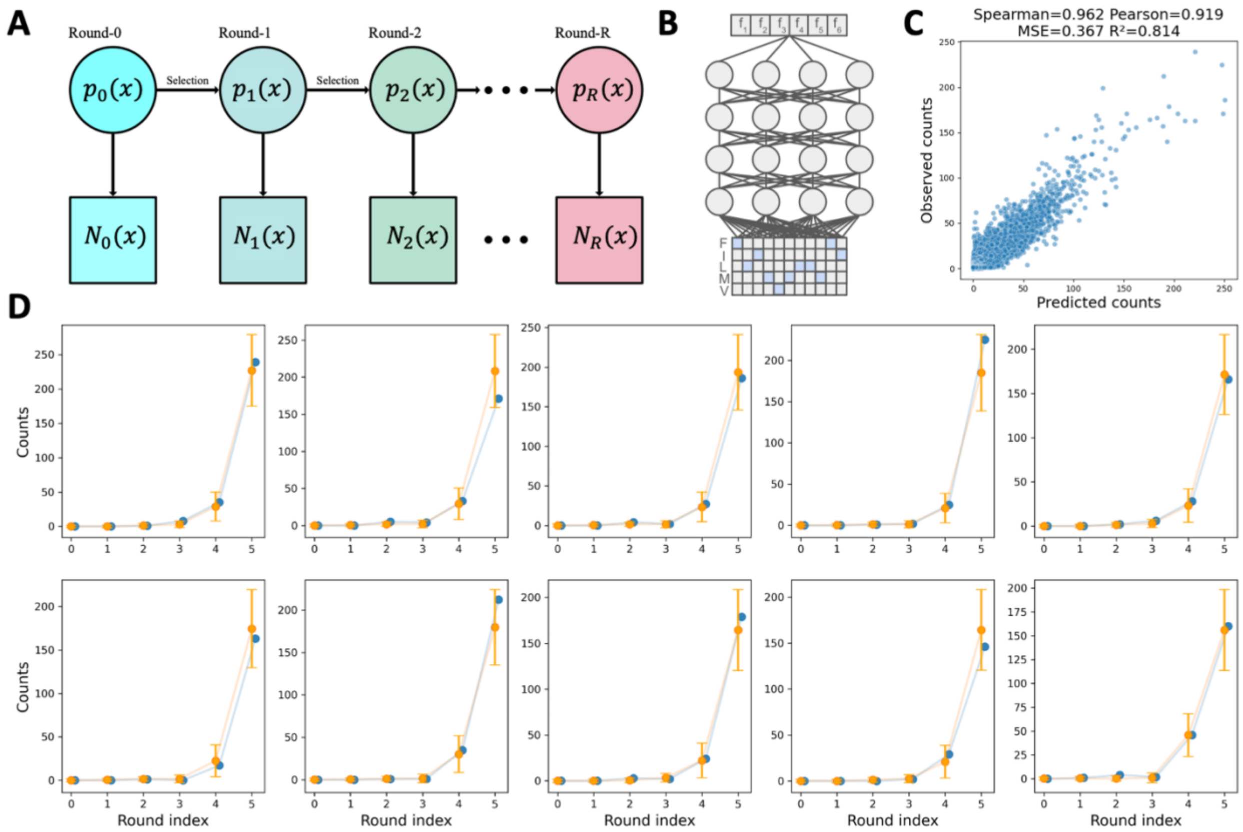

Fig. S1. Learning coevolution fitness landscape from multi-round selection experiments.
(**A**) Our selection probabilistic model is inspired by the experimental data-generating process consisting of two steps in each round: (i) a selection-induced update of the sequence distribution and (ii) an observation step where sequences are drawn from the current sequence distribution resulting in the sequencing read counts. Our model connects the fitness, which is related to the probability of a sequence to be selected in a specific round, with the counts. Round 0 denotes the pre-selection rounds. (**B**) Architecture of the fully connected neural network with four hidden layers used to parameterize the fitness function. It takes a one-hot encoded library sequence as input and can output the probability that a given sequence passes the selection at any given round, a quantity that is monotonically related to the fitness. Based on these probabilities one can predict the number of counts expected for a sequence in any round knowing the sampling budget (*i.e.*, the total number of counts observed in the experiment) in this round. (**C**) Comparison of predicted and experimentally observed read counts, in the final round of selection, for the sequences in the held-out dataset (20% of data not used to train the model). (**D**) Again, comparing predicted (orange dots) and experimentally observed (blue dots) read counts, now broken down by round, and only for the 10 strongest binders in the held-out dataset. Round 0 indicates the pre-selection library. Error bars

represent the 95% confidence intervals based on the multinomial distribution of counts learned by the model.

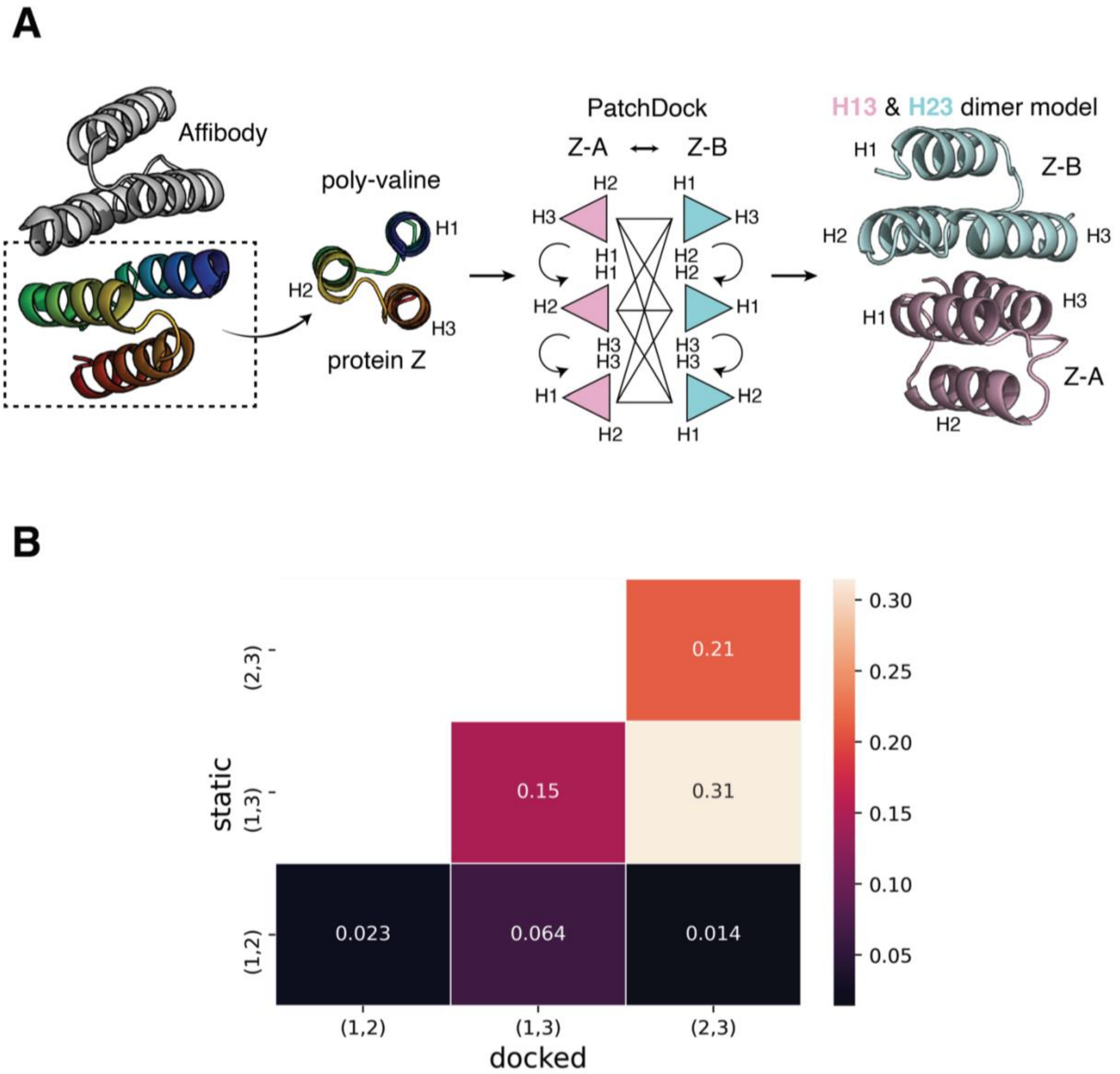

Fig. S2. Computational docking for coevolutionary library design.

(A) Schematic workflow of Z-domain-Z-domain docking and interface selection. A Z-domain derived from the Protein Z and affibody complex crystal structure (PDB: 1LP1) was minimized using Rosetta FastRelax while maintaining backbone constraints. A poly-valine variant was generated with RosettaRemodel to neutralize sequence-specific biases. This poly-valine Z-domain was docked against another poly-valine Z-domain using PatchDock, and models with interface areas greater than 1000 Å² were refined with Rosetta FastRelax. From these, the 25 lowest-energy models were manually inspected in PyMOL, with preference given to configurations involving

H1-H3 or H2-H3 contacts. Models were ranked based on Rosetta Energy Units (REU) and interface packing. The final model, featuring helices H13 and H23 interactions, was selected for subsequent sequence design and experimental validation.

(B) Heatmap showing preferred helix faces participating in interface of PatchDock models with an interface area > 1000Å. Color bar represents the fraction of designs adopting each binding mode among the 699 designs that meet this threshold.

A

	Z-A (H1+3)										Z-B (H2+3)								
	7	8	10	11	14	15	45	49	50	53	25	29	30	33	43	44	47	50	51
Z domain	K	E	Q	N	Y	E	L	K	K	D	E	A	F	S	N	L	E	K	L
M1 model	E	W	W	V	W	F	L	A	R	R	R	L	F	L	V	L	M	I	A
**Library**	A	X	W	X	X	X	X	A	A	A	I	X	X	X	X	X	X	I	A

X : M, F, L, I, V

B

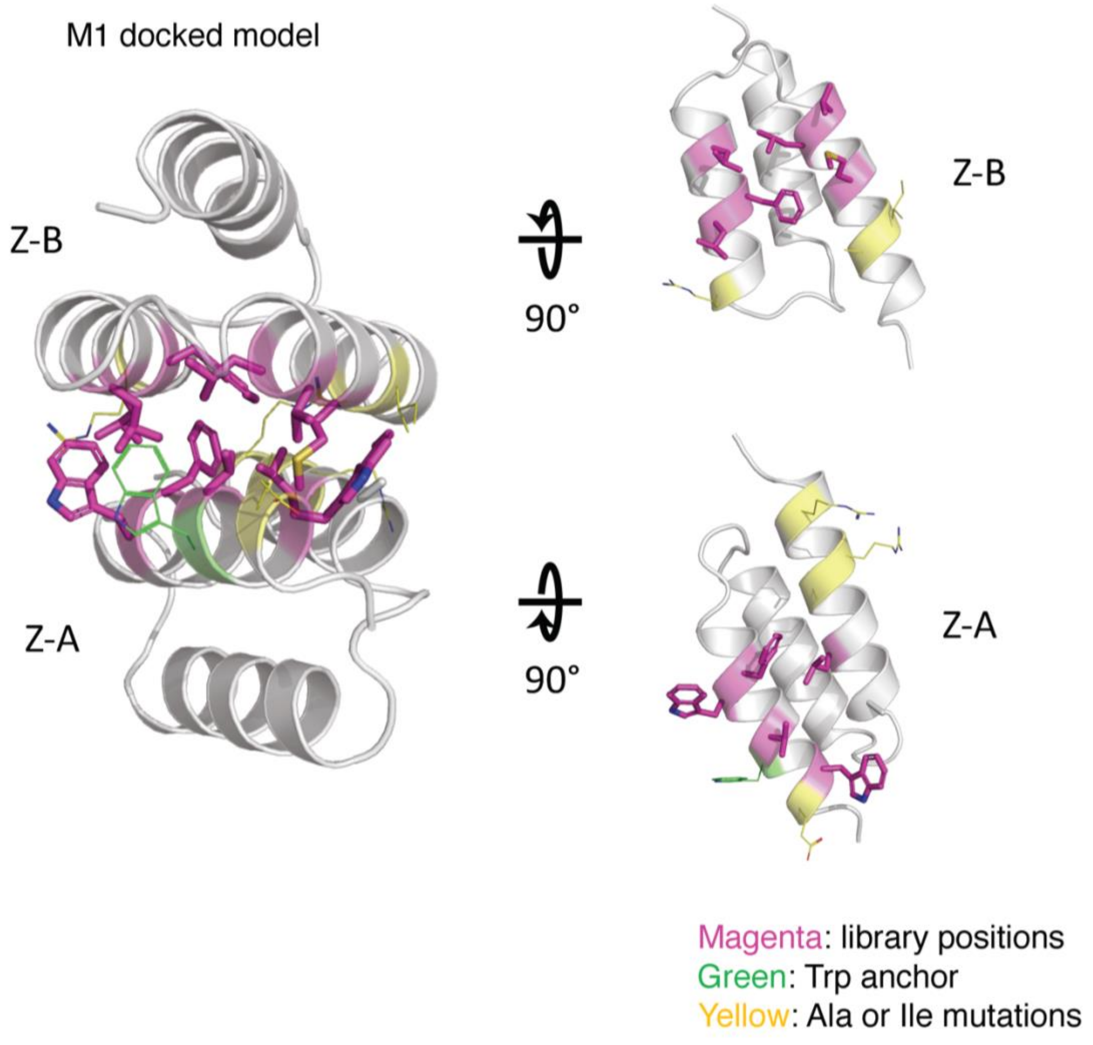

Fig. S3. Design of library positions based on the docked model.

(**A**) Library design and sequence diversification strategy. Sequences of the Z-domain (wild-type) and the M1 docked model (highest-ranked structure) at each library position are shown for chains Z-A (H1+3 helices) and Z-B (H2+3 helices). Library positions targeted for diversification are

marked in red (X), allowing substitutions with five hydrophobic residues (M, F, L, I, V). Positions constrained to alanine or isoleucine are highlighted in yellow. A conserved tryptophan residue, shown in green, was retained as a potential structural anchor to maintain interface stability.

(**B**) Structural representation of the M1 docked model. Side view (left) and open-book representation of the interface (right) highlight key design features. Library positions subjected to diversification are shown in magenta, while the conserved tryptophan residue is indicated in green. Positions converted to alanine or isoleucine to replace bulky residues are shown in yellow.

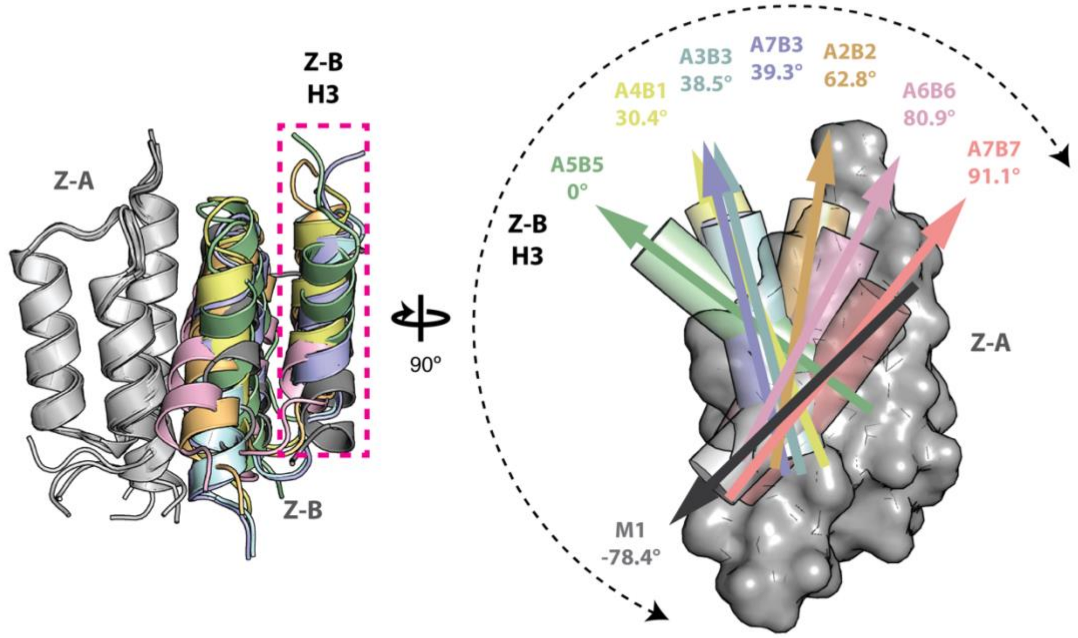

Fig. S4. Structural diversity and deviations in docking orientations of coevolved complexes compared to the docked model M1.

Superposition of seven coevolved complexes highlights variations in docking orientations relative to the initial computational docked model (M1). Full dimer structures (left) and Z-B H3 helices (right) are aligned and shown in distinct colors, emphasizing differences in docking orientations. Angular deviations are visualized relative to the H3 helix of Z-B in the reference A5B5 complex (green, 0°). Arrows represent the angular shifts in docking orientations, spanning 30.4° (A4B1) to 91.1° (A7B7). Notably, the initial docked model (M1) deviates significantly (-78.4°) from all evolved complexes, underscoring that none of the experimentally coevolved complexes recapitulates the M1 conformation.

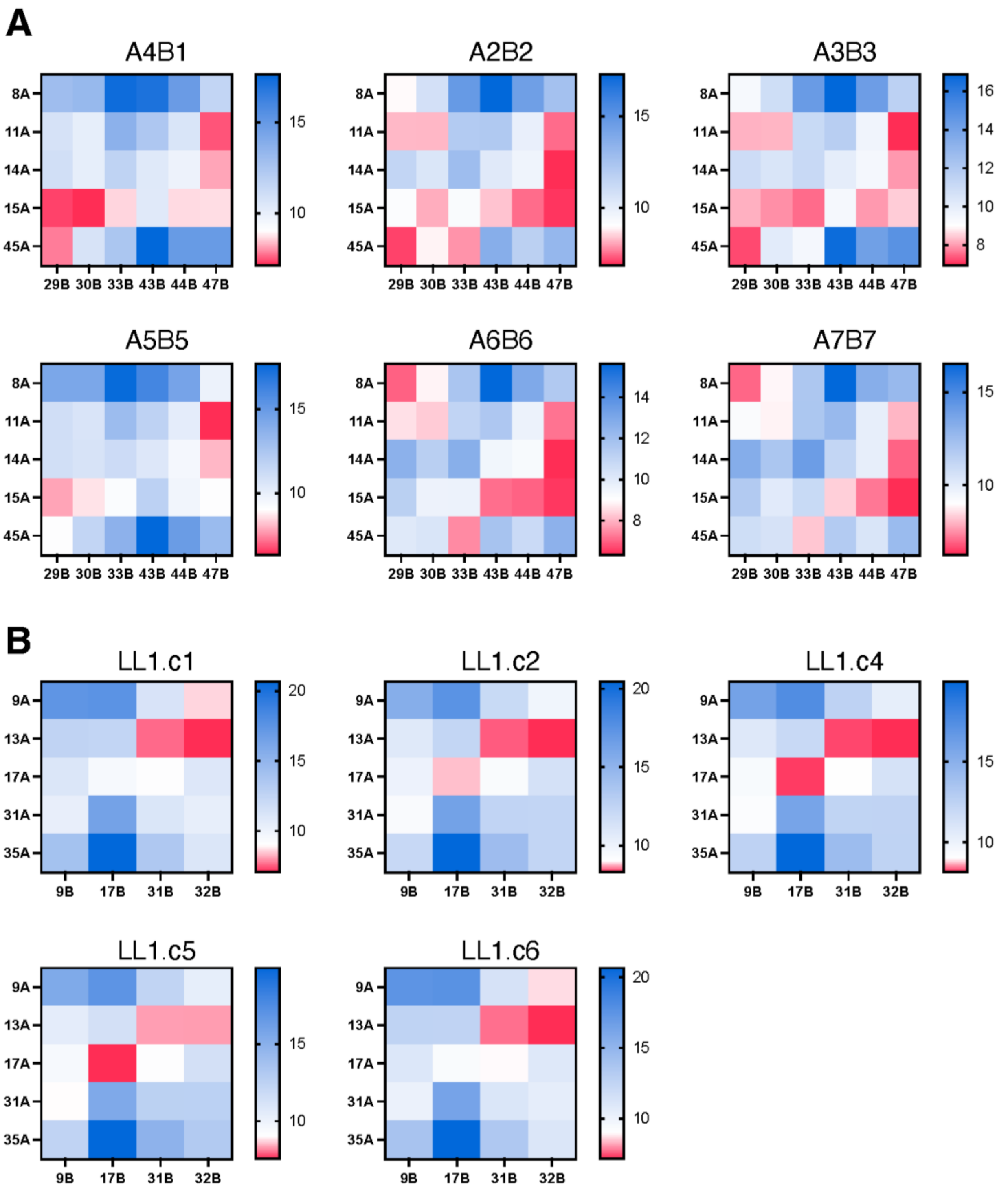

Fig. S5. Heatmaps of Cα-Cα distances between library positions in coevolved interfaces.
(A) Heatmaps showing Cα-Cα distances (in Å) between library positions in coevolved pairs (A4B1, A2B2, A3B3, A5B5, A6B6, A7B7) of synthetic interfaces. Rows correspond to residues in Z-A, and columns correspond to residues in Z-B. The color scale represents inter-residue distances, with

blue indicating longer distances and red indicating shorter distances. The midpoint of the color bar is set to 9 Å to emphasize residue pairs in close proximity that may form direct contacts.

(B) Heatmaps displaying Cα-Cα distances for selected pairs from natural interfaces (LL1.c1, LL1.c2, LL1.c4, LL1.c5, LL1.c6). The color scale and axes are consistent with panel (A).

These heatmaps illustrate the spatial proximity of residues at the library positions, providing structural insights into the physical organization of synthetic and natural interfaces. Synthetic interfaces exhibit greater variation in contact patterns, whereas natural interfaces display more conserved distance patterns.

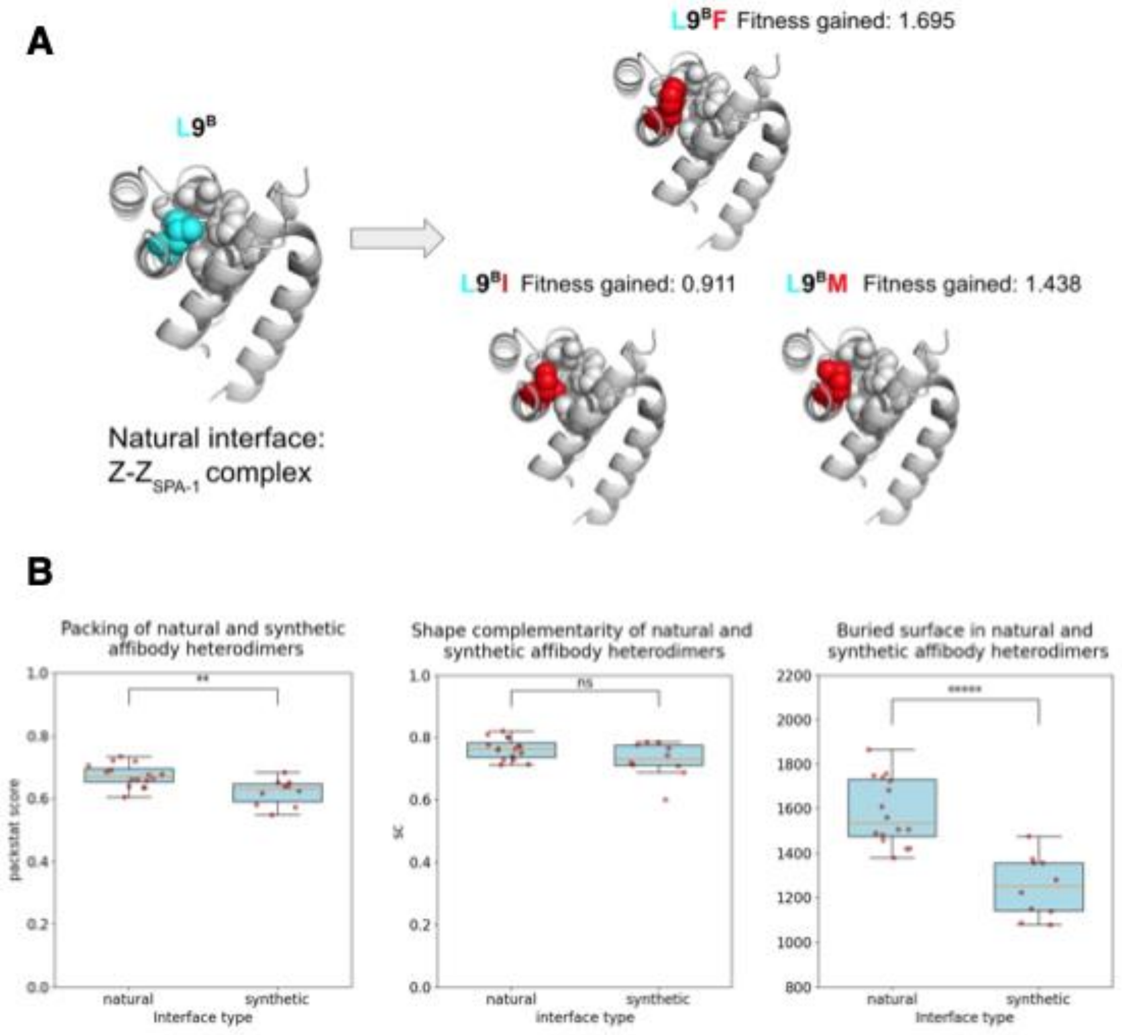

Fig. S6. (A) Left: crystal structure of the Z-Z_{SPA-1} complex (PDB ID: 1LP1) with sphere representation of $L9^B$ (in cyan) and its neighboring residues in the complex. Right: structural models of fitness-improved Z-Z_{SPA-1} mutants with sphere representation of mutated 9^B (in red) and its neighboring residues (in gray) in the complex. The structure models are built by mutating the amino acid at 9^B in the crystal structure of the Z-Z_{SPA-1} complex.

(B) Comparison of biophysical parameters between natural and synthetic affibody complexes. (left) Overall packing of complexes as measured by packstat. (middle) Shape complementarity of the interfaces as measured by sc (right) total buried solvent-accessible surface area in the interfaces. Significance of the difference is measured by two-tailed P test. (ns): $P > 0.05$; (**): $P < 0.01$; (*****): $P < 0.00001$

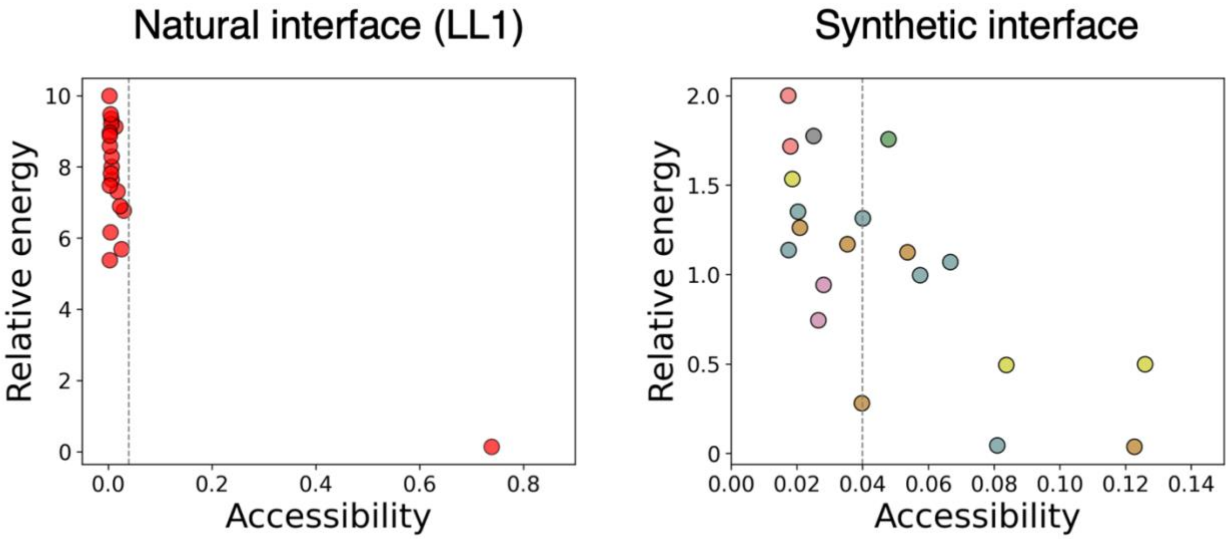

Fig. S7. Predicted relative energy and accessibility of the 20 most accessible energy wells for natural and synthetic interfaces.

Energy is defined as the negative fitness (*47*). We computed a relative energy for each sequence that represents an energy well by subtracting the energy of the strongest-binding sequence. Relative energies are comparable between the two plots, but absolute energy values are not. Vertical dashed lines mark accessibility of 0.04 for comparison between the two interfaces.

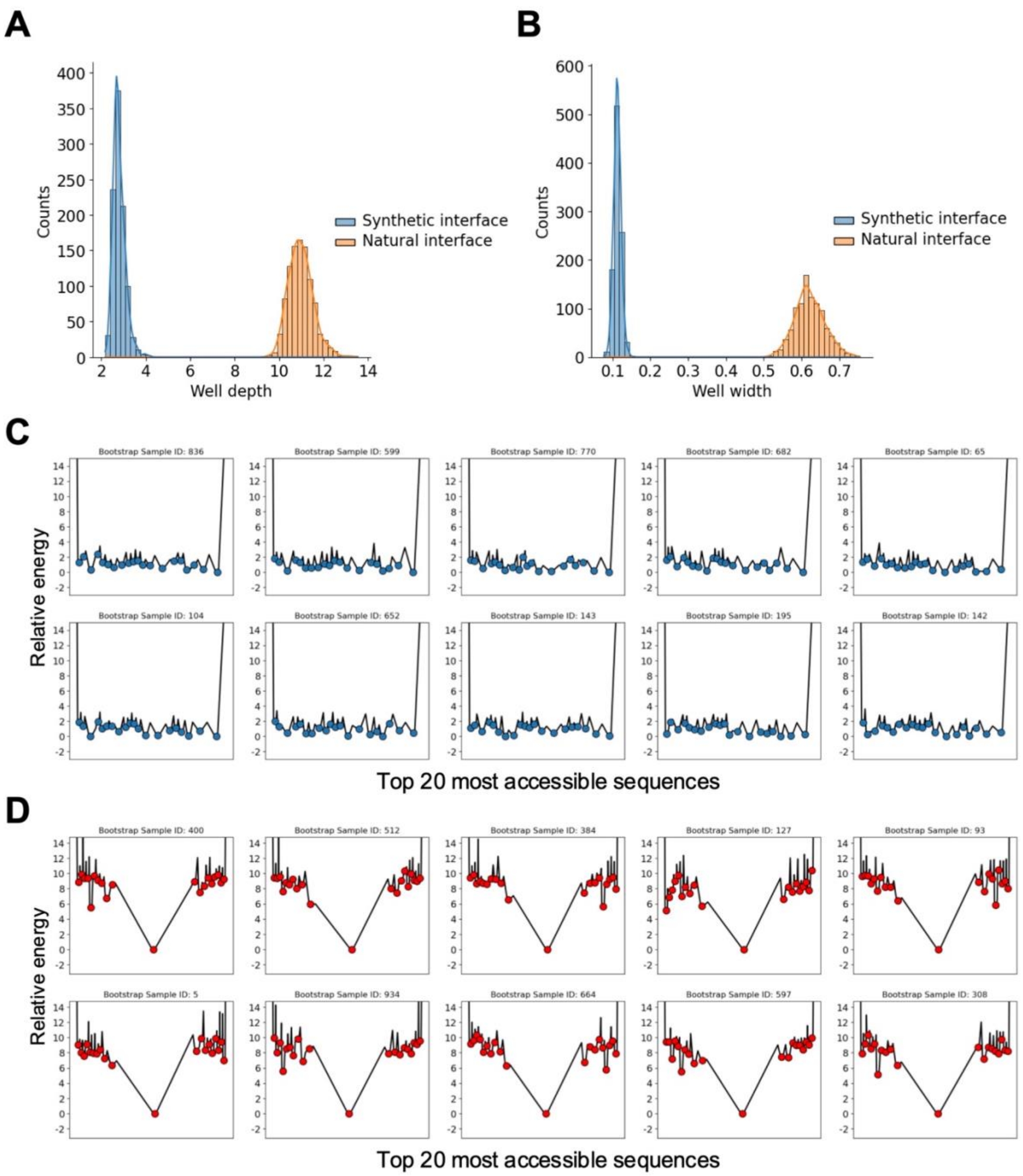

Fig. S8. Analysis of 1,000 bootstrapped NGS read data sets to determine statistical significance for differences in energy landscape geometries (*i.e.*, well depth and width, the latter corresponding to accessibility) between natural and synthetic interfaces. (A) Distributions of bootstrap well depths for the deepest energy well in the 20 most accessible wells in the synthetic interface (blue) and the most accessible energy well in the natural interface (orange). Each well depth in the distribution is obtained from an energy landscape estimated by SPM from a bootstrap sample of

NGS reads. (B) Distributions of bootstrap well widths (corresponding to accessibility) for the most accessible energy well (*i.e.*, widest) in the synthetic interface (blue) and the most accessible energy well in the natural interface (orange). Each width in the distribution is obtained from an energy landscape estimated by SPM from a bootstrap sample of NGS reads. (C and D) The same plots as in the main manuscript Figure 3F, here shown for analysis of 10 randomly chosen bootstrapped data sets. Recall, these plots show the relative energy landscapes of the 20 most accessible wells for the (C) synthetic interface and (D) the natural interface. To visualize the geometry of each landscape, we chose a random ordering of the 20 most accessible wells, with the exception of the natural interface which always centers the deepest energy well, using a random ordering for the rest.

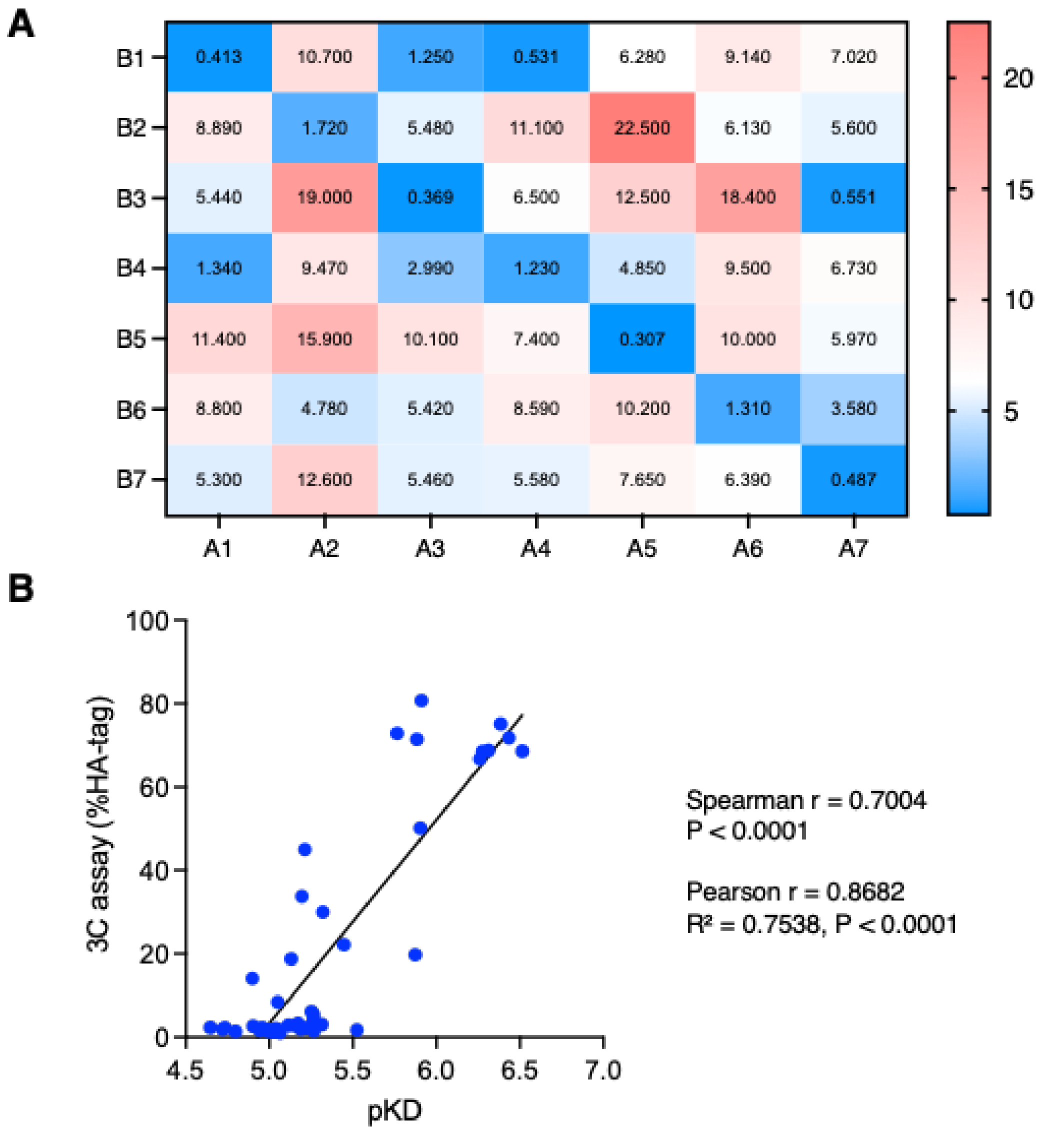

Fig. S9. Binding affinity of coevolved variants

(A) Solution affinities (K_D) of coevolved pairs from the specificity matrix (Fig. 4A) measured by surface plasmon resonance (SPR). Colors are centered at the median of all matrix values (6.3), with red indicating values above and blue indicating values below.

(B) Correlation between on-yeast cleavage-capture assay HA-tag readouts and binding affinity (pK_D) measured by SPR. (Spearman r = 0.7004; Pearson r = 0.8682, R^2 = 0.7538)

(C-D) Representative fitted SPR binding curves and corresponding K_D values for coevolved pairs. K_D values were determined using a steady-state model in Biacore evaluation software.

C

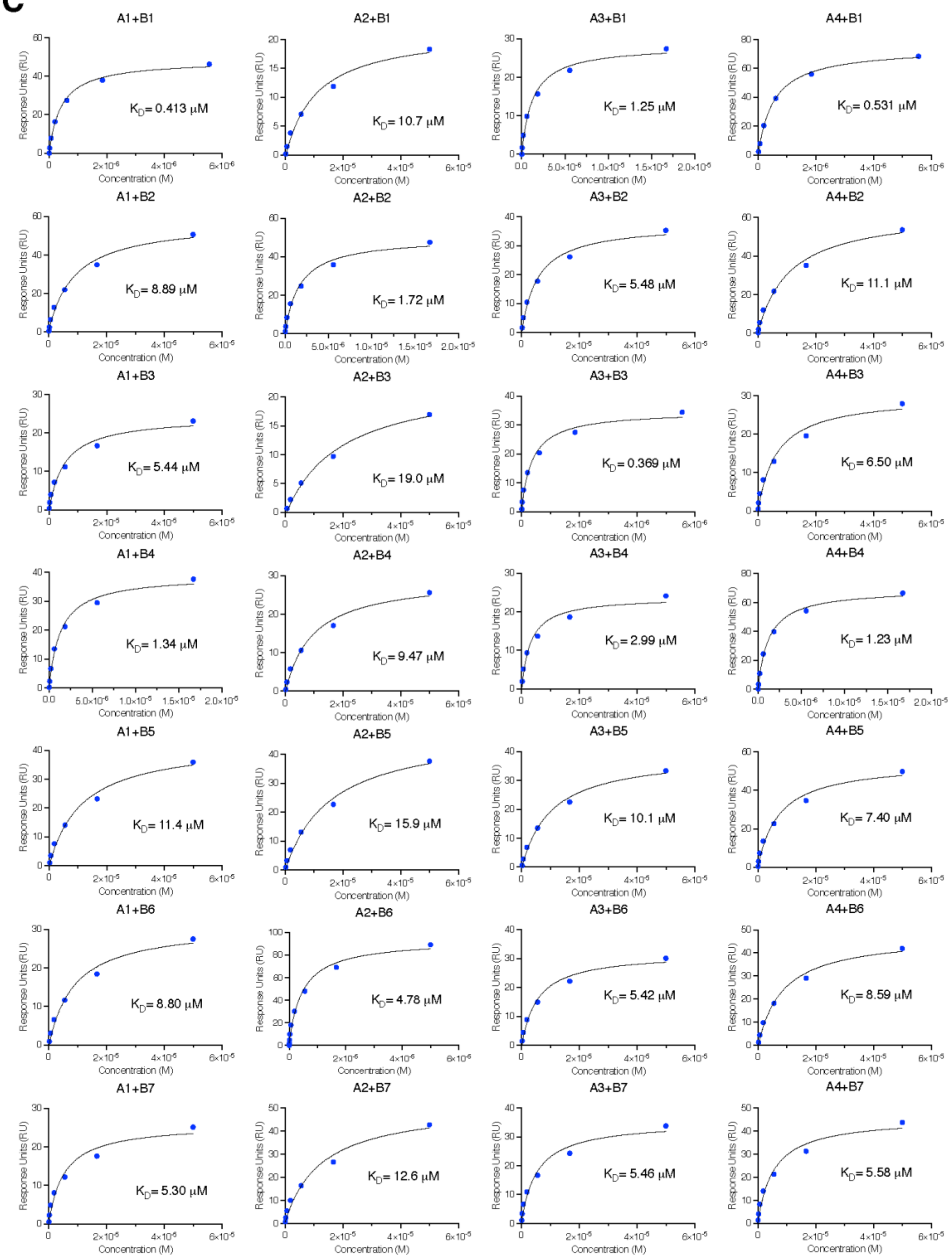
A1+B1
KD= 0.413 µM
A2+B1
KD= 10.7 µM
A3+B1
KD= 1.25 µM
A4+B1
KD= 0.531 µM
A1+B2
KD= 8.89 µM
A2+B2
KD= 1.72 µM
A3+B2
KD= 5.48 µM
A4+B2
KD= 11.1 µM
A1+B3
KD= 5.44 µM
A2+B3
KD= 19.0 µM
A3+B3
KD= 0.369 µM
A4+B3
KD= 6.50 µM
A1+B4
KD= 1.34 µM
A2+B4
KD= 9.47 µM
A3+B4
KD= 2.99 µM
A4+B4
KD= 1.23 µM
A1+B5
KD= 11.4 µM
A2+B5
KD= 15.9 µM
A3+B5
KD= 10.1 µM
A4+B5
KD= 7.40 µM
A1+B6
KD= 8.80 µM
A2+B6
KD= 4.78 µM
A3+B6
KD= 5.42 µM
A4+B6
KD= 8.59 µM
A1+B7
KD= 5.30 µM
A2+B7
KD= 12.6 µM
A3+B7
KD= 5.46 µM
A4+B7
KD= 5.58 µM
Response Units (RU)
Concentration (M)

D

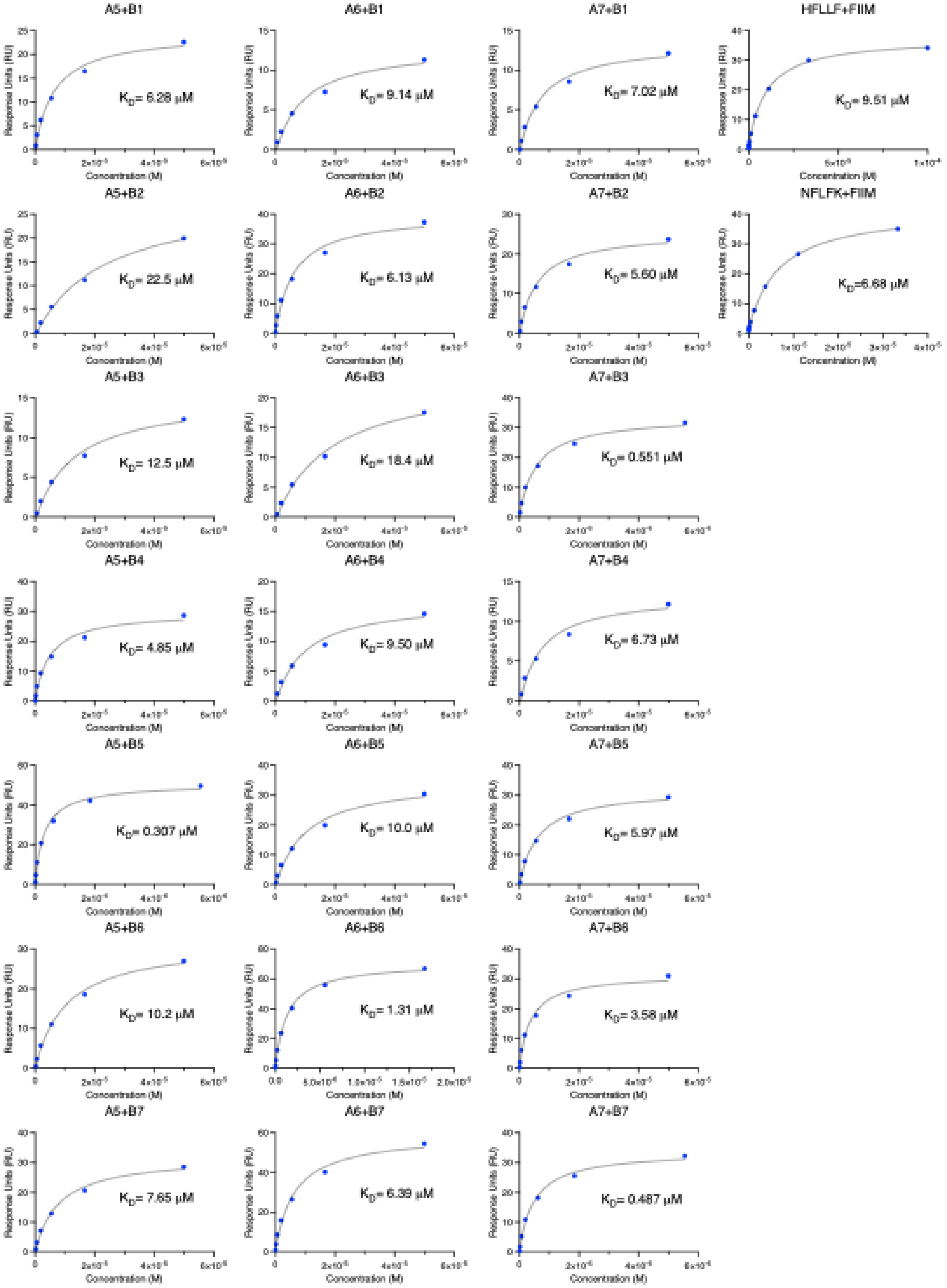
A5+B1
KD= 6.28 μM
A6+B1
KD= 9.14 μM
A7+B1
KD= 7.02 μM
HFLLF+FIIM
KD= 9.51 μM
A5+B2
KD= 22.5 μM
A6+B2
KD= 6.13 μM
A7+B2
KD= 5.60 μM
NFLFK+FIIM
KD=6.68 μM
A5+B3
KD= 12.5 μM
A6+B3
KD= 18.4 μM
A7+B3
KD= 0.551 μM
A5+B4
KD= 4.85 μM
A6+B4
KD= 9.50 μM
A7+B4
KD= 6.73 μM
A5+B5
KD= 0.307 μM
A6+B5
KD= 10.0 μM
A7+B5
KD= 5.97 μM
A5+B6
KD= 10.2 μM
A6+B6
KD= 1.31 μM
A7+B6
KD= 3.58 μM
A5+B7
KD= 7.65 μM
A6+B7
KD= 6.39 μM
A7+B7
KD= 0.487 μM
Response Units (RU)
Concentration (M)

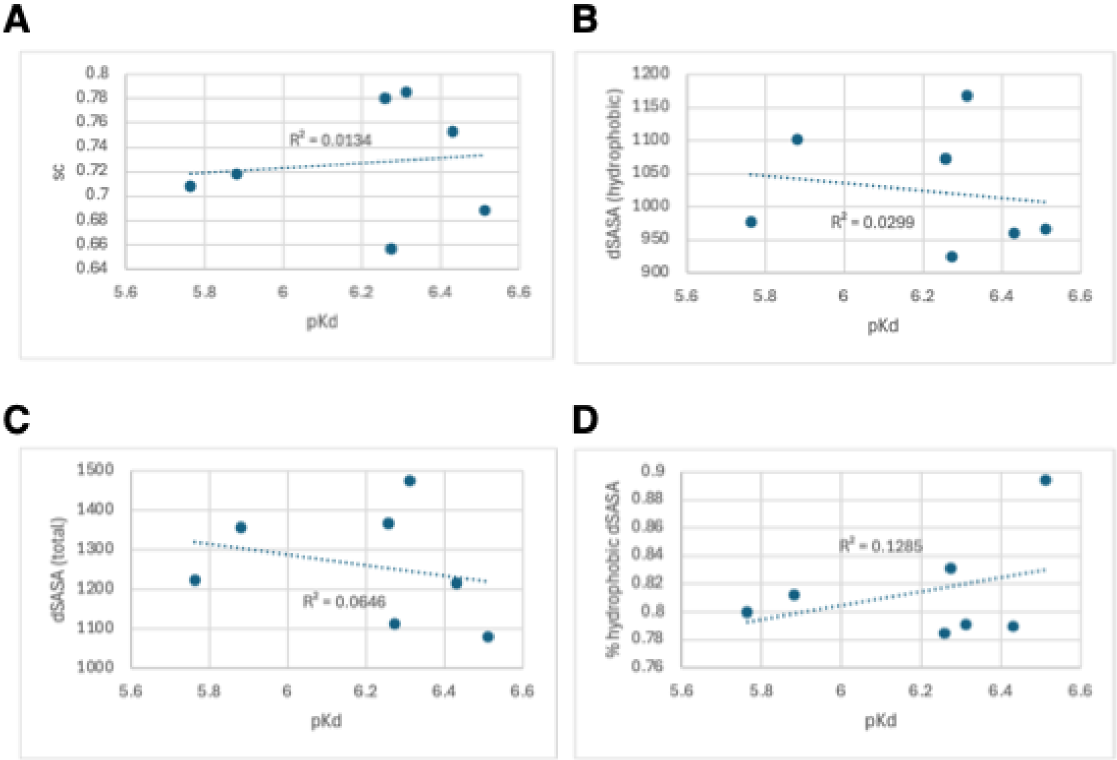

Fig. S10. Relationships between biophysical parameters and affinity for synthetic interfaces. No significant association was found between K_D and (A) shape complementarity (sc), (B) buried hydrophobic surface area, (C) total buried surface area, or (D) the hydrophobic fraction of total buried surface area for the crystal structures of our synthetic interface complexes. Mean of two is shown for structures that contain two complexes in the asymmetric unit.

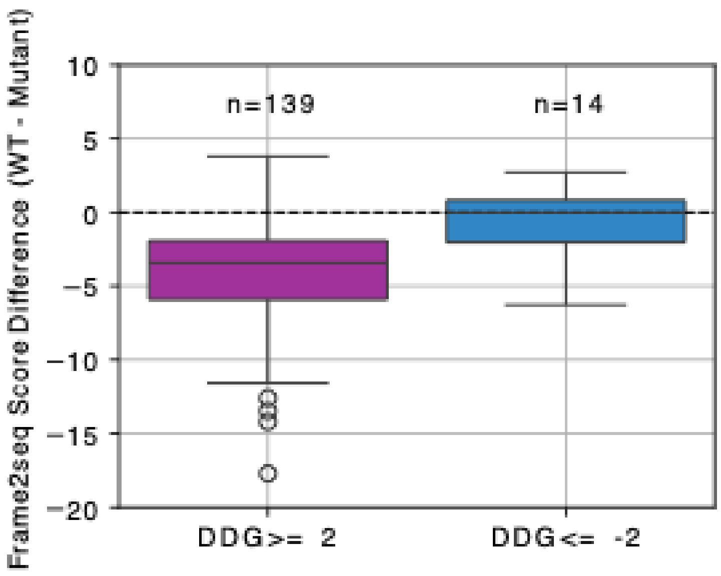

Fig. S11. Frame2seq distinguishes between mutations in protein-protein interfaces that have been experimentally measured to destabilize protein-protein binding (DDG>=2 kcal/mol, left) and mutations that have been experimentally measured to stabilize protein-protein binding (DDG<=-2 kcal/mol, right). Shown are boxplots of Frame2seq score differences between WT and mutant sequences for 153 cases from the Flex ddG dataset with strongly stabilizing or destabilizing mutations (*49*). Dashed line at y=0 indicates equal model preference between WT and mutated amino acids; negative score difference indicates Frame2seq prefers the wild-type interface. We note that while Frame2seq clearly identifies destabilizing mutations, it is more difficult to identify stabilizing mutations; this is a problem common to many prediction methods for protein-protein interfaces. n depicts number of variants. Boxes represent the interquartile range (IQR) of the data, with a solid line at the median, whiskers extending to 1.5 times the IQR, and points outside the whiskers are considered outliers.

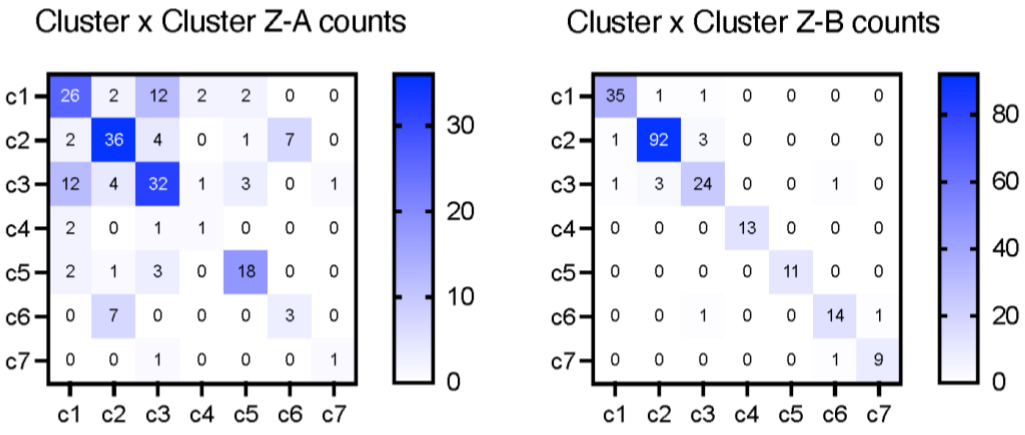

Fig. S12. Inter-cluster cross-reactivity analysis in round 5 NGS data

Cluster × Cluster heatmaps of Z-A (left) and Z-B (right) sequences showing the number of sequences with intra-cluster specificity or inter-cluster cross-reactivity across the seven SSN clusters (Fig. 3A). Of the 146 Z-A sequences analyzed, 29 (19.9%) displayed inter-cluster cross-reactivity, whereas only 7 of 205 Z-B sequences (3.4%) did so, indicating that cross-reactivity is limited and occurs primarily on the A chain. Beyond the two structurally characterized cases (A4B1 and A7B3), notable A-chain cross-reactivity was observed between clusters c1–c3 (n = 12) and c2–c6 (n = 7), consistent with the specificity matrix (Fig. 4A).

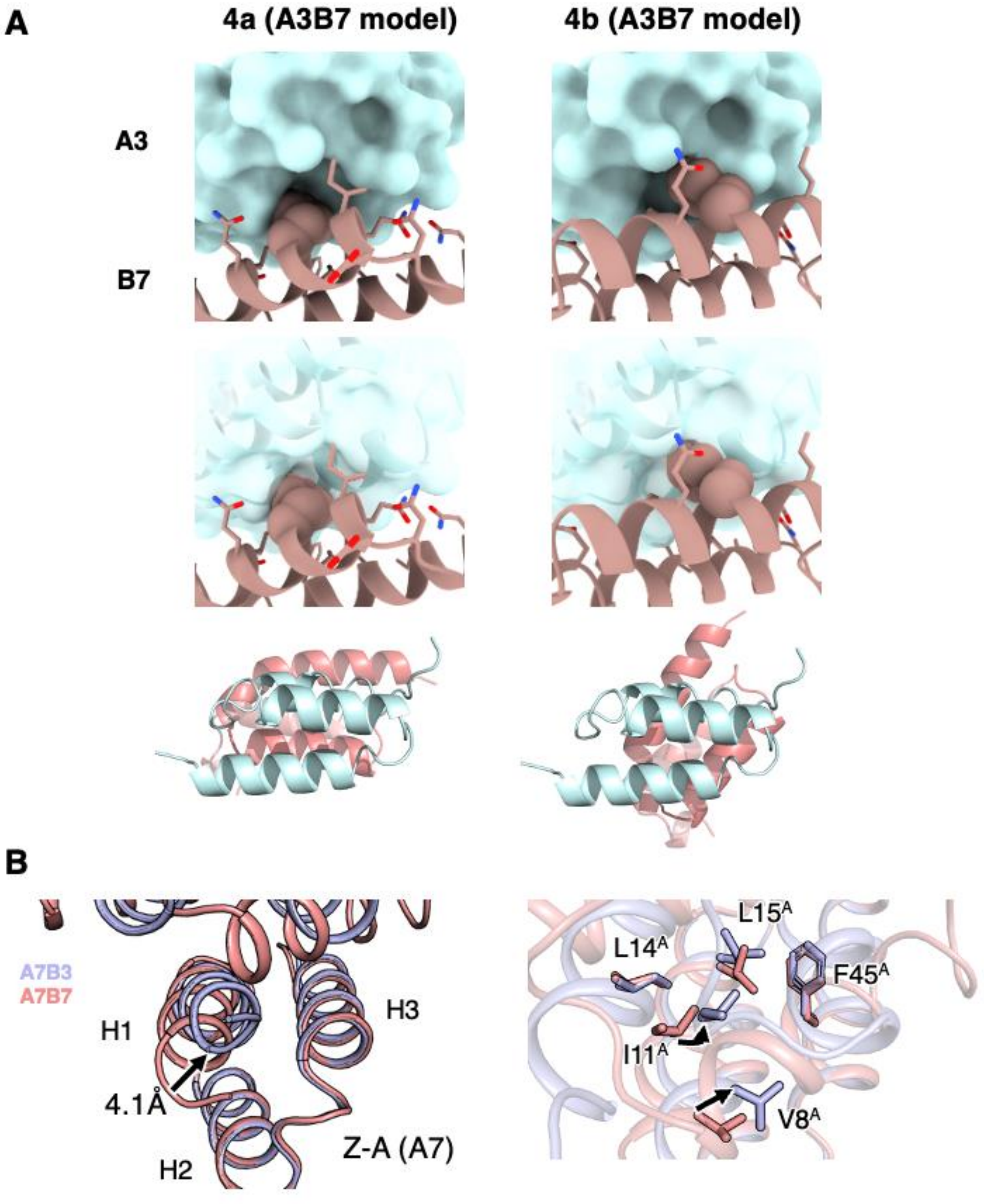

Fig. S13. Cross-reactivity between cluster 3 and 7 pairs.

(A) Structural models of the putative A3B7 complex in two alternative docking conformations (4a and 4b). Z-A subunits are shown as surface representations and Z-B subunits as cartoons, with

residue 29^B side chain atoms shown as spheres (top and middle). The Z-A chain (pale cyan) is shown as a surface representation, while the Z-B chain (dark salmon) is displayed as a cartoon. The middle panel shows a transparent surface for the Z-A subunit to more clearly visualize steric clashes at the interface. Bottom panels show top views of the models, illustrating the relative docking angles between the two chains. These models illustrate potential docking orientations of A3B7 based on structural alignment with A3B3 (4a) and A7B7 (4b) crystal structures. Both models suggest that A3B7 fails to form a stable interface due to steric clashes, in contrast to the docking flexibility observed in A7B3.

(B) Overlay of A7 structures from the A7B7 (salmon) and A7B3 (light blue) complexes reveals a helix shift (left). Close-up view of key interface residues (V8, I11, L14, L15, and F45) highlights side-chain reorientations that accommodate alternative docking modes (right).

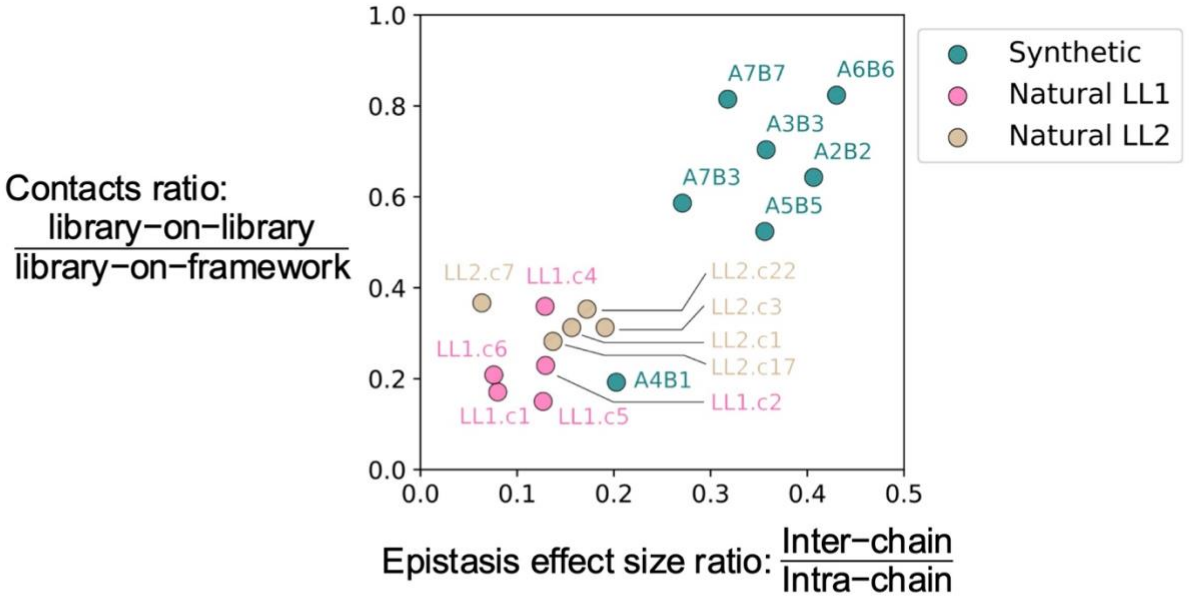

Fig. S14. Scatter plot of residue contact ratios and epistasis effect size ratios of the crystal structures.

For each crystal structure of synthetic interface or natural interface, the ratio of library-on-libary contacts over library-on-framework contacts is plotted against the ratio of average inter-chain epistasis effect size over average intra-chain epistasis effect size. We calculated Spearman rank correlation (correlation coefficient=0.719, with the p-value P=0.0008) and Pearson correlation (correlation coefficient=0.869, with the p-value P=0.0001) between the contacts ratios observed in the crystal structures and the epistasis effect size ratios computed from SPM. Each p-value was computed by a permutation test with 10,000 samples, wherein the contact ratio was randomly permuted so that it no longer corresponded to the epistasis effect size ratio for the same crystal structure.

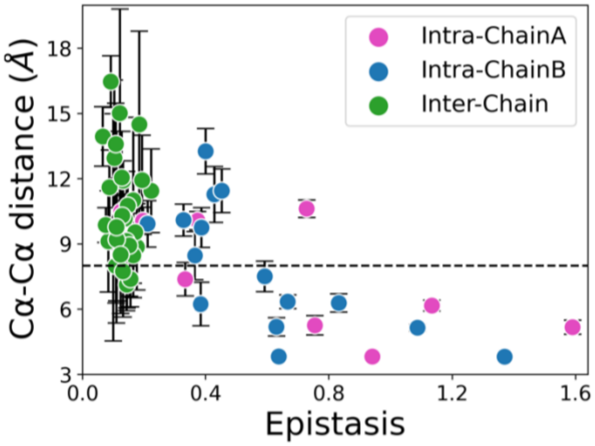

Fig. S15. The relationship between epistasis importance and structures.

Scatter plot of two metrics: (i) Cα-Cα distances between library residue pairs in the crystal structures and (ii) pairwise epistasis importance for those same residue pairs. Pink, blue and green dots denote intra-ChainA, intra-ChainB and inter-chain residue pairs respectively. Spearman correlations: -0.550 (overall), -0.660 (Intra-ChainA), -0.659 (Intra-ChainB) and -0.212 (Inter-Chain).

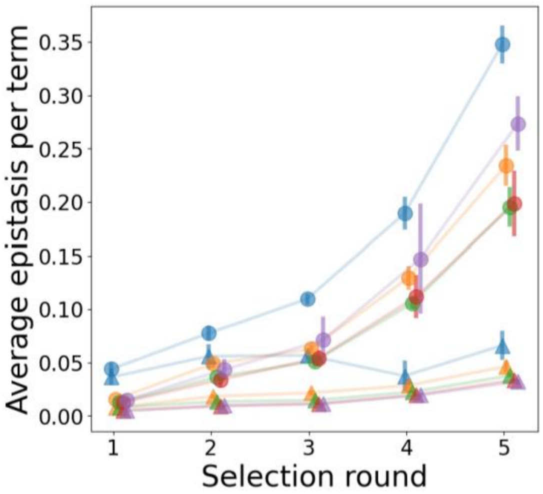

Fig. S16. Average intra-chain and intra-chain epistasis importance for each round of selection experiments of synthetic interface.

Dots denote the intra-chain epistatic terms and triangles denote the inter-chain epistatic terms. The colors indicate different orders of epistasis: blue (second order), orange (third order), green (fourth order), red (fifth order) and purple (sixth order). Error bars represent the 95% confidence intervals based on five independently sampled datasets from bootstrapping the sequencing data.

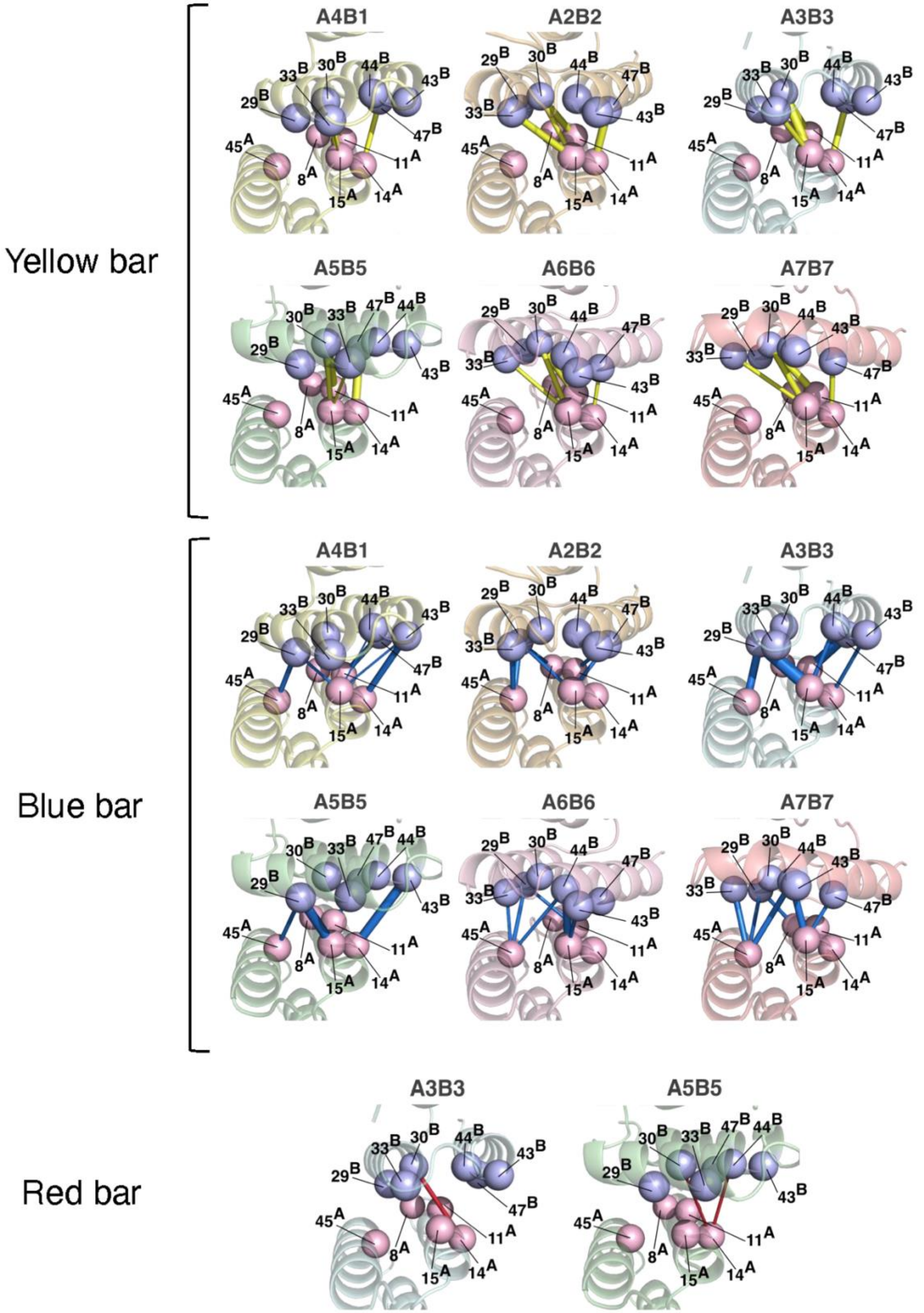

Fig. S17. Visualization of pairwise epistasis terms on cluster representative structures

Spheres highlight library positions where light pink spheres belong to chain A and light blue spheres belong to chain B. Bar thickness represents epistasis importance as computed using Frame2seq. Yellow: Epistasis term is within the top 10 strongest terms as ranked by both SPM and Frame2seq on every cluster structure. Blue: Epistasis term is within the top 10 strongest terms as ranked by Frame2seq on that particular cluster structure. Red: Epistasis term is within the top 10 strongest terms as ranked by both SPM and Frame2seq on that particular cluster structure.

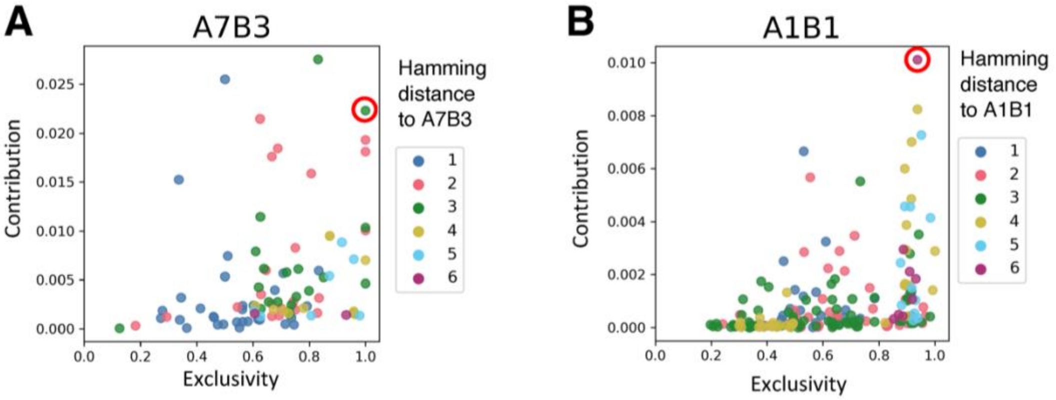

Fig. S18. Identification of seed sequences for crystal structures A7B3 and A1B1.

(A) Seed sequences of A7B3. For each seed, its contribution towards A7B3 is plotted against its exclusivity towards A7B3. The seeds are colored based on the Hamming distance of their sequences from the sequence of A7B3. Seed highlighted by red circle is further analyzed. (D) Seed sequences of A1B1. For each seed, its contribution towards A1B1 is plotted against its exclusivity towards A1B1. The seeds are colored based on the Hamming distance of their sequences from the sequence of A1B1. Seed highlighted by red circle is further analyzed.

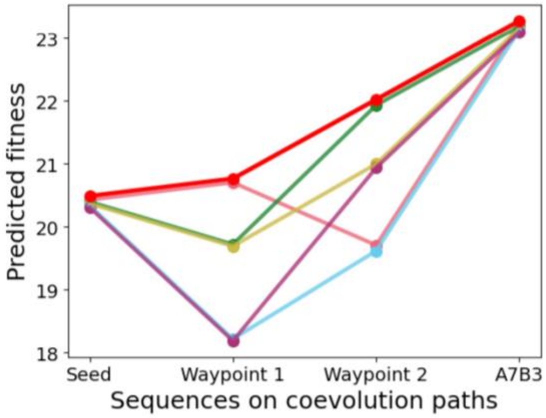

Fig. S19. Predicted fitness for the seed, A7B3 and the waypoints on all the coevolutionary paths.

The red line indicates the only path where each mutation step results in the improvement of the predicted fitness.

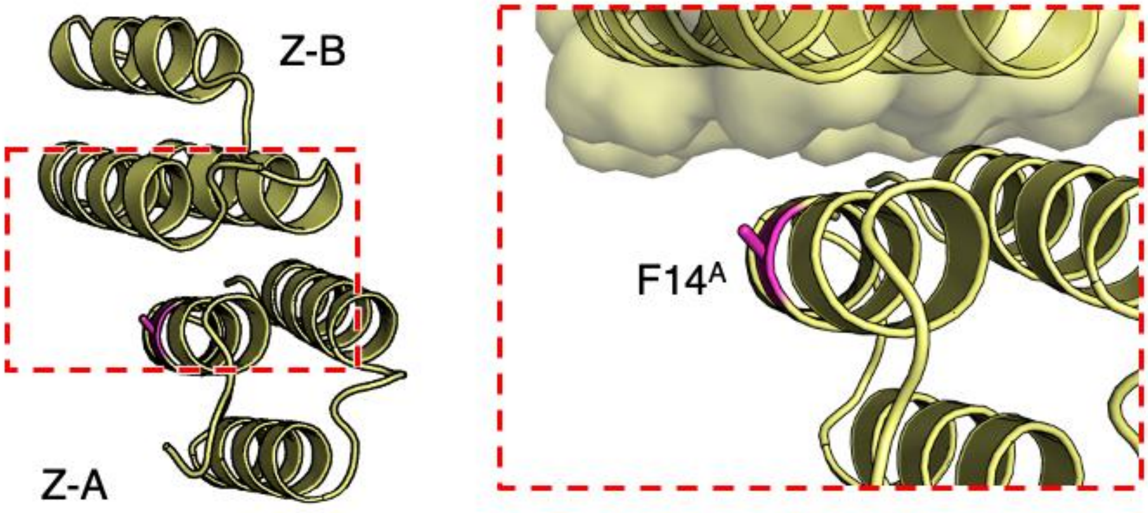

Fig. S20. Structural rationale for selecting A4B1 as the representative of cluster 1.

The left panel shows the overall structure of the A4B1 complex, highlighting the interface region (red dashed box). The right panel provides a close-up view of the interface, focusing on residue $F14^{A}$ (magenta) in Z-A. This residue represents the single mutation difference between A4B1 and A1B1. $F14^{A}$ is located outside the binding interface, where its side chain exhibits flexibility, leading to weak electron density and reduced structural constraints. Due to this positioning, the mutation does not significantly impact the interface interaction, making A4B1 structurally equivalent to A1B1 for representing cluster 1.

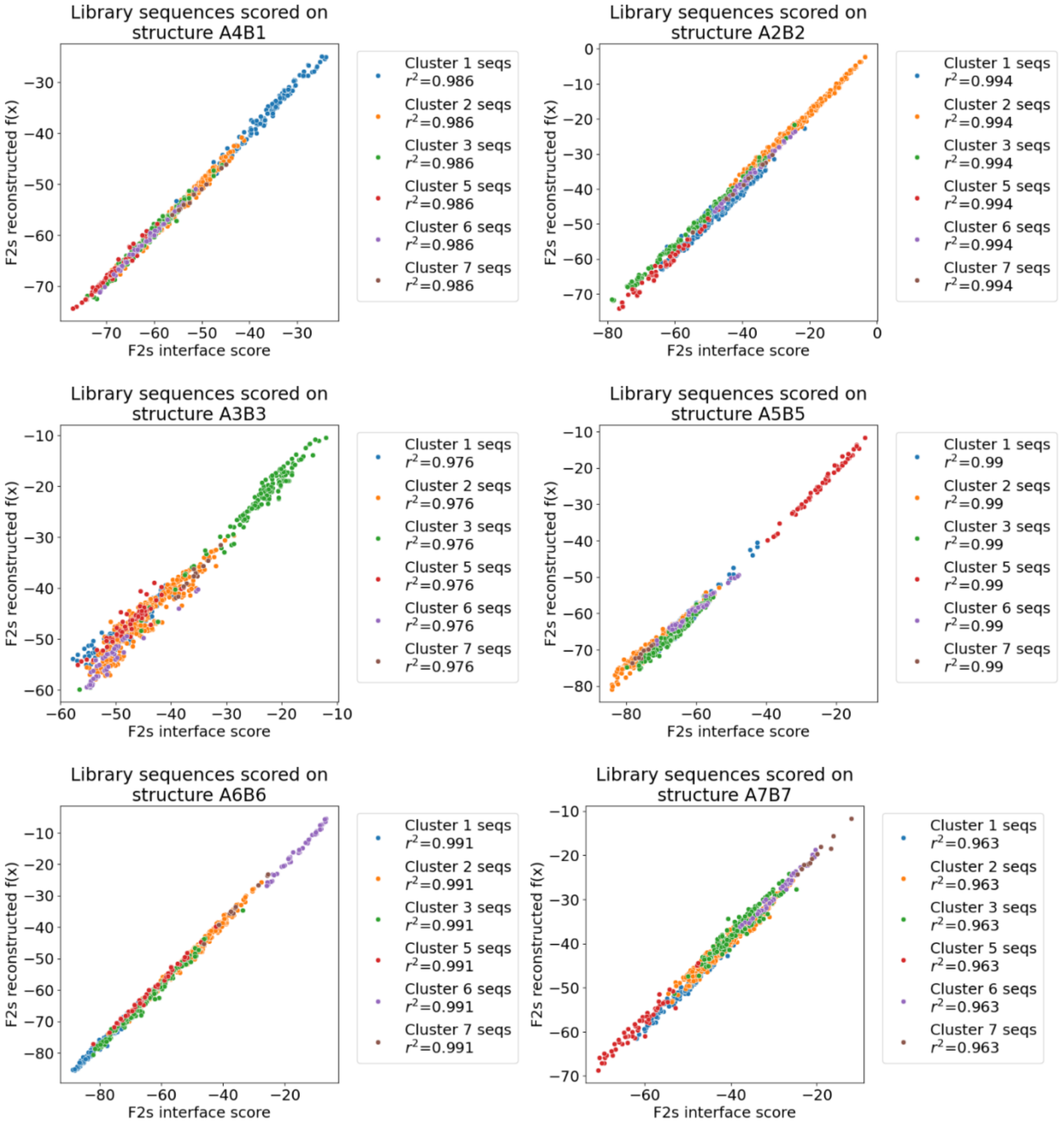

Fig. S21. Frame2seq reconstructed fitness function vs Frame2seq interface score on library sequences when conditioned on cluster backbone structures.

Only library sequences whose cluster assignment have a corresponding crystal structure are shown.
